# Comparative analysis of dielectric, shear mechanical and light scattering response functions in polar supercooled liquids

**DOI:** 10.1038/s41598-021-01191-9

**Published:** 2021-11-12

**Authors:** K. L. Ngai, Z. Wojnarowska, M. Paluch

**Affiliations:** 1 Dipartimento di Fisica, CNR-IPCF, Università di Pisa, Largo Bruno Pontecorvo 3, 56127 Pisa, Italy; 2 Institute of Physics, University of Silesia in Katowice, 75 Pułku Piechoty 1A, 41-500 Chorzow, Poland

**Keywords:** Physics, Condensed-matter physics, Phase transitions and critical phenomena, Structure of solids and liquids

## Abstract

The studies of molecular dynamics in the vicinity of liquid–glass transition are an essential part of condensed matter physics. Various experimental techniques are usually applied to understand different aspects of molecular motions, i.e., nuclear magnetic resonance (NMR), photon correlation spectroscopy (PCS), mechanical shear relaxation (MR), and dielectric spectroscopy (DS). Universal behavior of molecular dynamics, reflected in the invariant distribution of relaxation times for different polar and weekly polar glass-formers, has been recently found when probed by NMR, PCS, and MR techniques. On the other hand, the narrow dielectric permittivity function ε*(f) of polar materials has been rationalized by postulating that it is a superposition of a Debye-like peak and a broader structural relaxation found in NMR, PCS, and MR. Herein, we show that dielectric permittivity representation ε*(f) reveals details of molecular motions being undetectable in the other experimental methods. Herein we propose a way to resolve this problem. First, we point out an unresolved Johari–Goldstein (JG) β-relaxation is present nearby the α-relaxation in these polar glass-formers. The dielectric relaxation strength of the JG β-relaxation is sufficiently weak compared to the α-relaxation so that the narrow dielectric frequency dispersion faithfully represents the dynamic heterogeneity and cooperativity of the α-relaxation. However, when the other techniques are used to probe the same polar glass-former, there is reduction of relaxation strength of α-relaxation relative to that of the JG β relaxation as well as their separation. Consequently the α relaxation appears broader in frequency dispersion when observed by PCS, NMR and MR instead of DS. The explanation is supported by showing that the quasi-universal broadened α relaxation in PCS, NMR and MR is captured by the electric modulus M*(*f*) = 1/ε*(*f*) representation of the dielectric measurements of polar and weakly polar glass-formers, and also M*(*f*) compares favorably with the mechanical shear modulus data G*(*f*).

## Introduction

Dielectric spectroscopy, an experimental technique to study the relaxation and diffusion of materials, has a long and glorious history in scientific research. One may trace it back to 1854 of the measurements of electrical relaxation of alkali ions in the Leyden jar (a glass) by Kohlrausch^1^ which led to the fractional exponential correlation function attributed to him,

1 $${\varphi }_{K}\left(t\right)=\text{exp}\left[-{\left(\frac{t}{{\tau }_{\alpha }}\right)}^{{\beta }_{K}}\right],$$

where *τ*_*α*_ is the structural α-relaxation of glass-formers and *β*_*K*_ is a fractional exponent. Another notable milestone of dielectric spectroscopy was the theoretical description of polarization phenomena of polar molecules by Debye in 1913^2^, and tested the description by the dielectric response of several monohydroxy alcohols reported in his 1929 book ‘Polar Molecules’^3^. One example of the modern developments is the dielectric measurement of glycerol by Davidson and Cole^4^. They proposed a relaxation function to represent the frequency dependence of the dielectric susceptibility ε*(*f*). Since then, dielectric spectroscopy has become widely used to study molecular structural relaxation and ionic conductivity relaxation in various materials^5^. Nowadays, the advance in instrumentation makes it possible to measure dielectric response over 18 decades of frequency^6^. The abundance of dielectric relaxation data accumulated to the present time enables the observation of the widely different dynamics in diverse materials and glass-formers belonging to the same class.

For materials with significant dipole moment, dielectric spectroscopy has the sensitivity to observe faster processes, including the secondary relaxation and nearly constant loss originating from caged molecular dynamics. It is the technique used by Johari and Goldstein to reveal the presence of secondary relaxations in many glass-formers and make the critical discovery that the secondary relaxation is present even in rigid molecules^7^. This is followed by another finding that each glass-former has a secondary relaxation that bears an inseparable connection in properties with the primary structural α-relaxation^8–10^. To distinguish this secondary mode from secondary relaxations not having these properties, it is called the Johari-Goldstein (JG) β-relaxations^11^. An example of the properties is the anti-correlation of *β*_*K*_ with the separation between the JG β-relaxation and the α-relaxation given by the logarithm of the ratio of their relaxation times, log(*τ*_*α*_/*τ*_*JG*_) at the glass transition temperature *T*_*g*_^12,13^. This anti-correlation is supported theoretically by the Coupling Model (CM) via the approximate relation^14^,

2 $$\text{log}{\tau }_{\alpha }-\text{log}{\tau }_{JG}\approx (1-{\beta }_{K})\text{log}{(\tau }_{\alpha }/{t}_{c}),$$

where *t*_*c*_ is the onset of classical chaos and has the value of 1 to 2 ps for molecular glass-formers determined by quasielastic neutron scattering.

A recent application of dielectric spectroscopy to van der Waals molecular glass-formers has found that the width of the α-loss peak near the glass transition temperature *T*_*g*_ is strongly anti-correlated with the polarity of the molecule. The larger the dielectric relaxation strength Δ*ε*(*T*_*g*_) or the glass-former is more polar, the narrower is the α-loss peak (anti-correlation) and larger is the Kohlrausch exponent *β*_*K*_ in Eq. (1) (correlation)^15^. This remarkable property was explained by the contribution from the dipole–dipole interaction potential to the attractive part of the intermolecular potential, making the resultant potential more harmonic. The consequence is ${\beta }_{K}^{DS}$ (i.e. *β*_*K*_ from dielectric spectroscopy) and the narrowing of the α-relaxation increasing rapidly with the dipole moment *μ* and Δ*ε*(*T*_*g*_). Subsequent tests of the correlation have repeatedly confirmed it^16–19^.

Let us combine this correlation of ${\beta }_{K}^{DS}$ with Δ*ε*(*T*_*g*_) with the anti-correlation between ${\beta }_{K}^{DS}$ and log(*τ*_*α*_/*τ*_*JG*_) at *T*_*g*_ mentioned in the above and given by Eq. (2). The combination strongly suggests the presence of the JG β-relaxation and its separation from the α-relaxation becomes smaller in glass-formers having narrower α-relaxation or larger ${\beta }_{K}^{DS}$. This fact is essential for anyone who raises the issue with the correlation of ${\beta }_{K}^{DS}$ with Δ*ε*(*T*_*g*_) to recognize. As we shall show in this paper that this fact becomes relevant for the issue raised in 2020 by Körber et al.^19^, who question the relevance of the correlation of ${\beta }_{K}^{DS}$ with Δ*ε*(*T*_*g*_) established from dielectric spectroscopy (DS) by the contrasting results from photon correlation spectroscopy (PCS), Fabry–Perot interferometry, and nuclear magnetic resonance relaxometry. In a number of the polar molecular glass-formers having narrow α-relaxation or large ${\beta }_{K}^{DS}$ and with large Δ*ε*(*T*_*g*_) they show the α-relaxations probed by PCS and NMR are much broader. Remarkably the widths of the α-relaxations from PCS and NMR varies weakly among the polar liquids with *β*_*K*_ falling within the range of 0.58 ± 0.06. Actually, only a few polar liquids were presented by Körber et al. to show the contrast between the larger value of ${\beta }_{K}^{DS}$ compared to the smaller value of ${\beta }_{K}^{PCS}$. We pick the best two cases; one is from glycerol with ${\beta }_{K}^{DS}$ = 0.69 vs. ${\beta }_{K}^{PCS}$ = 0.52, and the other one is from phenolphthalein dimethylether (PDE) with ${\beta }_{K}^{DS}$ = 0.76 vs. ${\beta }_{K}^{PCS}$ = 0.55. More examples are given in this paper to show the contrast between the larger ${\beta }_{K}^{DS}$ and the smaller values of ${\beta }_{K}^{G}$ from shear modulus G* measured by mechanical relaxation in a number of polar glass-formers.

It should be mentioned even earlier in 2019 that Gainaru had found practically the same spectral shape from viscoelastic measurements of polar and nonpolar van der Waals, hydrogen-bonded, and ionic liquids^20^. Relying on the common spectral shape emerging from the combination of different susceptibility results, he explained the correlation between ${\beta }_{K}^{DS}$ and Δ*ε*(*T*_*g*_) of the dielectric *α* process of van der Waals liquids. Also, Pabst et al.^21,22^ found that the light scattering spectra of the same systems almost perfectly superimpose and show a generic line shape of the structural relaxation, following the ω^−1/2^-dependence at high frequencies. In dielectric spectra, the generic behavior found by other techniques holds only for nonpolar systems with a low dipole moment. The much narrower dielectric loss peak in highly polar molecules was rationalized in Pabst et al. by the presence of a intense Debye-like contribution from cross-correlation, which overrides the generic broader structural relaxation aaumed.

Körber et al.^19^ seem to imply that the correlation of ${\beta }_{K}^{DS}$ with Δ*ε*(*T*_*g*_) from dielectric spectroscopy is not general because it does not hold when the dynamics is probed by the other spectroscopies. From their results that the Kohlrausch exponent *β*_*K*_ for a given substance is method independent except dielectric spectroscopy (DS), Körber et al.^19^ seem to imply that the dynamics from DS is not fundamental. At the International Dielectric Society Meeting on 30 September 2020, Rössler cited from a referee report of the paper by Körber et al.^19^ the following remark: “After reading this paper I couldn’t help but wonder if we could have saved 20 years of viscous liquid research if we had not spent so much on dielectric spectroscopy”. These are profound implications that could undermine the verity of the voluminous amount of data taken by DS and theoretical interpretations over more than a century, which is currently the technique commonly used by numerous researchers in glass-forming materials around the world. This unsettling status of DS imposed by the above remark needs to be addressed and reexamined independently. This is the purpose of the present paper by going deeper and broader into the experimental data. We performed shear mechanical modulus G*(*f*) measurements in several glass-formers and collected G*(*f*) and PCS data of other glass-formers from the literature to compare with dielectric relaxation data. Moreover, we explain why the Kohlrausch exponent *β*_*K*_ observed by G*(*f*), PCS and NMR are smaller than ${\beta }_{K}^{DS}$ by DS in highly polar glass-formers with large ${\beta }_{K}^{DS}$ and Δ*ε*(*T*_*g*_). The key to the explanation is the presence of the JG β-relaxation lying close by the α-relaxation according to Eq. (2) because of the larger value of ${\beta }_{K}^{DS}$ close to 1. It has low dielectric strength compared to the dominant α-relaxation. The two factors combined make it unresolved in the dielectric spectra. It does not affect the main part of the frequency dispersion of the α-relaxation and the value of ${\beta }_{K}^{DS}$ in the fit by the Fourier transform of the Kohlrausch function. On the other hand, when measured by the other spectroscopies, the relaxation strength of the α-relaxation relative to the JG β-relaxation is substantially reduced. Consequently, the width of α-relaxation becomes broader due to the overlap with the relatively higher level of the JG β contribution, resulting in smaller values of the Kohlrausch exponent *β*_*K*_ in the other spectroscopies than ${\beta }_{K}^{DS}$. Thus the larger ${\beta }_{K}^{DS}$ of highly polar molecules found by DS truly reflects the frequency dispersion and the dynamics of the α-relaxation of the polar molecular glass-formers, whereas the results are muddled in the spectra measured by the other methods.

## Cause of the broadening of the α-relaxation of polar glass-formers when probed by G*, PCS, and NMR

Following the first paper in 1998, the presence of a secondary relaxation having strong connections with the α-relaxation (with properties including Eq. (2)) has been found in many glass-formers^23^. A notable property of the JG β-relaxation is the pressure dependence of its relaxation time *τ*_*JG*_. When considering both pressure *P* and temperature *T* dependence, Eq. (2) takes the form

3 $$\text{log}{\tau }_{\alpha }\left(P,T\right)-\text{log}{\tau }_{JG}\left(P,T\right)\approx \left[1-{\beta }_{K}\left(P,T\right)\right]\left[\frac{\text{log}{\tau }_{\alpha }\left(P,T\right)}{{t}_{c}}\right].$$

A general property found by DS is the co-invariance of $\text{log}{\tau }_{\alpha }\left(P,T\right)-\text{log}{\tau }_{JG}\left(P,T\right)$ and ${\beta }_{K}\left(P,T\right)$ to variations of *P* and *T* while keeping ${\tau }_{\alpha }(P,T)$ constant. The term JG β-relaxation was chosen for such secondary relaxation to distinguish it from other and usually intramolecular secondary relaxations. The JG β-relaxation is predicted to be present in all glass-formers since the omnipresent primitive relaxation of the CM is a part of the distribution of processes in the JG β-relaxation, and the primitive relaxation time *τ*_0_ is approximately equal to the most probable JG β-relaxation time *τ*_*JG*_, i.e., *τ*_*JG*_ ≈ *τ*_0_^24^. This approximate relation was one of the criteria commonly used to check if a resolved secondary relaxation is the JG β-relaxation or not. For those polar and highly polar glass-formers with larger ${\beta }_{K}^{DS}$, the JG β-relaxation is not resolved because $(\text{log}{\tau }_{\alpha }-\text{log}{\tau }_{JG})$ according to Eq. (2) is small, and hence it is not well separated from the dominant α-relaxation. Nevertheless, the dielectric loss data cannot be accounted entirely by the Fourier transform of a Kohlrausch function. There is an excess loss on the high-frequency flank of the Kohlrausch fit, and in addition, an excess wing shows up at higher frequencies in some cases such as propylene carbonate, glycerol^25,26^, quinaldine^27^, and picoline^28^. The excess wing should be distinguished from the nearly constant dielectric loss *ε*″(*f*) ∝ *f*^*−λ*^ with λ small and positive, which is due to loss while molecules are mutually caged by the anharmonic intermolecular potential. There are several facts supporting that the excess loss and the excess wing come from the unresolved JG β-relaxation, although this is still not universally accepted. (1) Long-term aging experiments performed on propylene carbonate, propylene glycol and glycerol^29,30^ show the excess wing was transformed to a broad shoulder making the JG β-relaxation partially resolved. (2) The relation in the frequency of the excess loss/excess wing to the α-loss peak remains unchanged with variations of *P* and *T* while the α-loss peak frequency is kept constant in propylene carbonate, aroclor (polychlorinated biphenyls), salol, and other polar and highly polar glass-formers, in accord with the property of JG β-relaxation given by Eq. (3). (3) The separation in frequency between the excess wing and the α-loss peak agrees with that calculated by the right-hand-side of Eq. (2) using the dielectric ${\beta }_{K}^{DS}$ for ${\beta }_{K}$ therein. (4) Highly polar glass-formers with larger ${\beta }_{K}^{DS}$ such as quinaldine^31^, picoline^32^, and cyanobenzene^33^ have no resolved secondary relaxation at all, and methyltetrahydrofuran (MTHF)^34,35^ and diethyl phthalate (DEP)^36^ have a non-JG secondary relaxation. A JG β-relaxation belonging to all of these glass-formers was resolved by mixing with a higher *T*_*g*_ non-polar component. These experiments indicate that the JG β-relaxation is present in these highly polar glass-formers but located too close to the dominant α relaxation and not resolved. The root cause is the more harmonic and hence weaker intermolecular interaction resulting from the dipole–dipole interaction contribution to the attractive part of intermolecular potential^15^. The resultant more harmonic and weaker intermolecular potential is consistent with the larger values of ${\beta }_{K}^{DS}$ for the α-relaxation observed by dielectric relaxation and molecular dynamics simulations^23^.

From the narrative given above, we postulate the presence of an unresolved JG β-relaxation in polar and highly polar glass-formers having dielectric strength small compared to the α-relaxation. It shows up as the excess loss/excess wing on the high-frequency flank of the narrow dielectric α-loss peak. Notwithstanding, it does not alter the frequency dispersion of the α-relaxation, and thus the dielectric Kohlrausch exponent ${\beta }_{K}$ truly reflects the dynamic heterogeneity and cooperativity of the α-relaxation. With this done, we are ready to suggest the cause of the dramatic broadening when probed by shear modulus (SM), PCS and NMR. A priori, there is no reason to expect the responses of the JG β-relaxation relative to the α-relaxation observed in susceptibility by DS is exactly preserved when probed by any of the other methods simply because the correlation functions are different. Moreover, dielectric susceptibility ε* is compliance and shear mechanical G* is the modulus, and the difference is another reason for expecting a change. As we shall show by actual dielectric data, the much larger strength Δ*ε*(*T*_*g*_) of the α-relaxation relative to Δ*ε*_*JG*_(*T*_*g*_) of the unresolved JG β-relaxation in polar glass-formers shown in permittivity becomes much reduced when represented in electric modulus, resulting in broadening of the former by the latter. Therefore a heuristic explanation of the broader α-relaxation observed by the other methods than DS in polar glass-formers is a decrease of the relaxation strength of the α-relaxation relative to that of the JG β-relaxation. The reduction of the relaxation strength of the α-relaxation when probed by the other methods with little or no change of the JG β-relaxation is plausible. This is because the cooperative many-body α-relaxation is more sensitive to change of correlation function and/or change from compliance to modulus than the JG β-relaxation. This heuristic explanation needs to be tested by experimental data. The results are presented in the following sections.

## Experimental verifications

We have proposed a heuristic explanation of why the narrow dielectric α-loss peak with large ${\beta }_{K}^{DS}$ at temperatures near *T*_*g*_ of polar glass-formers becomes a broader loss peak with smaller *β*_*K*_ when probed by the other methods. In supporting this explanation, we have made new dielectric and shear modulus measurements of several glass-formers and also have collected and reanalyzed previously obtained data. All the polar glass-formers showing the difference in α-relaxation dispersion of dielectric and PCS considered by Körber et al. are covered here. Additionally, we added more cases not included in their paper. The results are reported below, and the explanation is reiterated wherever deemed necessary.

### Highly polar glass-formers

We have mentioned that the ratio of the relaxation strengths of the α and JG β relaxations of polar glass-formers with large Δ*ε* = (*ε*_0_ − *ε*_∞_) can be reduced when converted to electric modulus representation, resulting in a broader modulus loss peak. The most direct test is to compare ε*(*f*) with the electric modulus M*(*f*) = 1/ε*(*f*). Actually, ε*(*f*) and its time-domain correspondent *ε*(*t*) should be referred to as dielectric retardation. The true dielectric relaxation is the modulus M*(*f*) and *M*(*t*). *M*(*t*) can be directly determined by measuring the time decay of the electric field *E*(*t*)–*M*(*t*) under constant charge conditions, as demonstrated by Wagner and Richert in poly(vinylacetate) and cresolphthaleine-dimethyl-ether (KDE), and hence also M*(*f*) is obtained directly after Fourier transformation^37,38^. Thus one can obtain M*(*f*) either indirectly from 1/ε*(*f*) or directly from *M*(*t*) after Fourier transform, and the result should be the same as demonstrated by Wagner and Richert.

#### Cresolphthaleine-dimethyl-ether (KDE)

**Figure 1:**
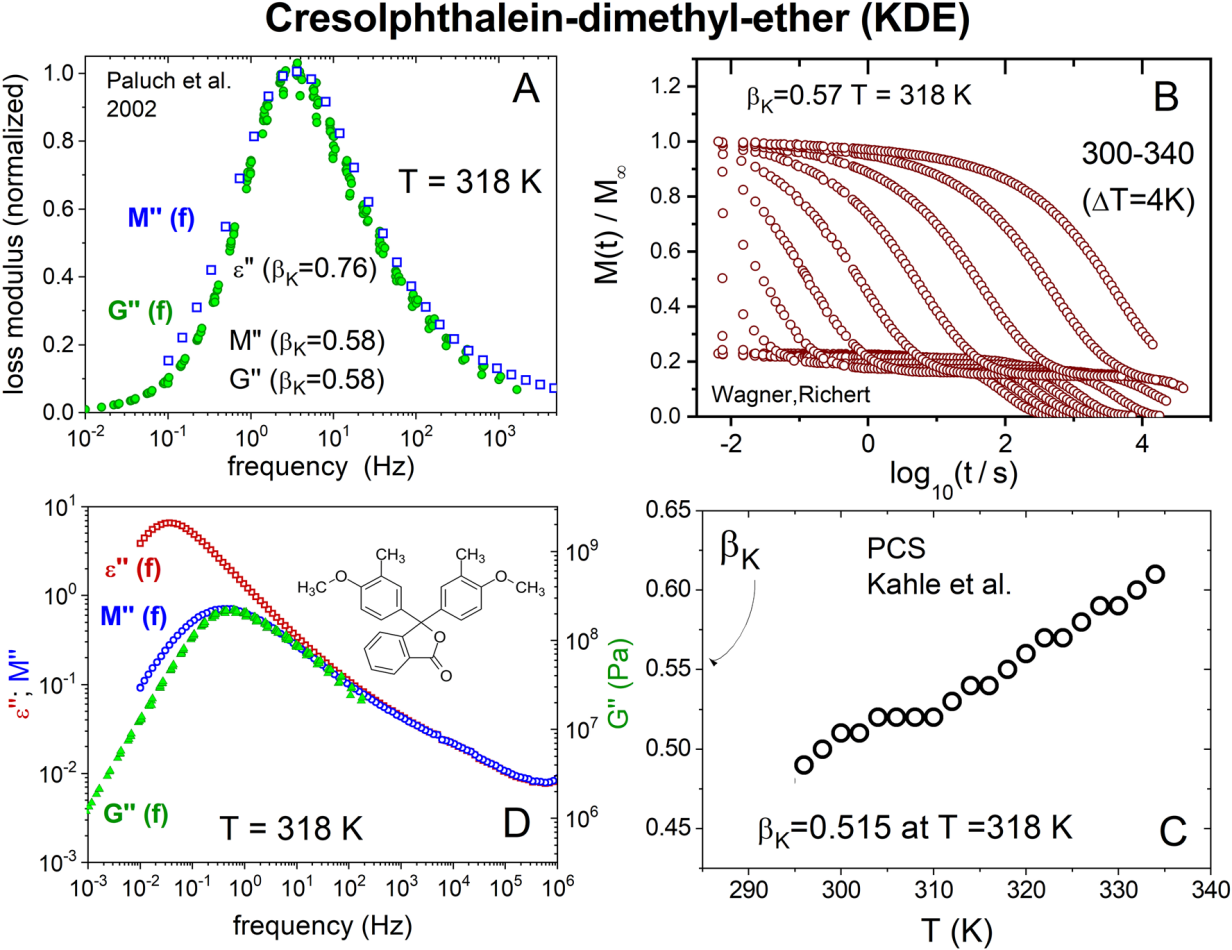
Collection of data of cresolphthaleine-dimethyl-ether (KDE). (**A**) Shows the frequency dispersions of *M*″(*f*) and the scaled *G*″(*f*) are nearly the same and broader than that found in *ε*″(*f*), and explains why the Kohlrausch exponents ${\beta }_{K}^{M}$ = 0.58 and ${\beta }_{K}^{G}$ = 0.58 are smaller than ${\beta }_{K}^{DS}$ = 0.76. (**B**) Shows the *M*(*t*) data of KDE from Richert and Wagner^38^ having time dependence well described by the Kohlrausch function with ${\beta }_{K}^{M}$ = 0.57. (**C**) Shows the exponents ${\beta }_{K}^{PCS}(T)$ of the light scattering intensity autocorrelation functions from Kahle et al.^40^ and assume the value of ${\beta }_{K}^{PCS}$ = 0.51 at 318 K. (**D**) Shows the *ε*″(*f*), and the vertically shifted data of *M*″(*f*), and scaled *G*″(*f*) data of KDE at 318 K from Ref.^39^ showing the large strength of the α-relaxation in *ε*″(*f*) is significantly reduced in *M*″(*f*).

The fact that the same M*(*f*) is obtained either indirectly from 1/ε*(*f*) or directly from *M*(*t*) after Fourier transform is verified by the several independent studies of KDE, a highly polar glass-former having dielectric Δε = 20 and ${\beta }_{K}^{DS}$ = 0.75. The *M*(*t*) data of KDE from Richert and Wagner has time dependence well described by the Kohlrausch function (Eq. (1)) with ${\beta }_{K}^{M}$ = 0.57 (see Fig. 1B), while M*(*f*) obtained from 1/ε*(*f*) by Paluch et al. was fitted by the Fourier transform of nearly the same Kohlrausch function with ${\beta }_{K}^{M}$ = 0.58 (see Fig. 1A)^39^. The data of *G*″(*f*) at the same temperature show a slightly narrower peak, and the Kohlrausch function used to fit has ${\beta }_{K}^{G}$ = 0.58. The time dependence of the VH light scattering intensity autocorrelation functions from Kahle et al.^40^ was fitted to the Kohlrausch function. The exponents ${\beta }_{K}^{PCS}(T)$, shown in Fig. 1C, decrease with temperature and assume the value of ${\beta }_{K}^{PCS}$ = 0.51 at 318 K. The correlation function of PCS is the second order Legendre polynomial and the susceptibility ${\chi }_{PCS}^{,,}(f)$ is a compliance and not modulus. Nevertheless, it is much broader than *ε*″(*f*) and its ${\beta }_{K}^{PCS}$ = 0.55 is significantly smaller than ${\beta }_{K}^{DS}$ = 0.76. More comparison of ${\chi }_{PCS}^{,,}(f)$ data from PCS with *ε*″(*f*) and *M*″(*f*) of polar glass-formers will be given later.

In Fig. 1D we compare the *ε*″(*f*), *M*″(*f*), and *G*″(*f*) data of KDE at 318 K. Compared with ε*(*f*), it is well known that M*(*f*) is shifted to higher frequencies by a factor of about ε_s_/ε_∞_. To observe the decrease of the relaxation strength of the α-relaxation with little or no change of the JG β-relaxation in *M*″(*f*) and *G*″(*f*), we shift the *M*″(*f*) data vertically as well the scaled *G*″(*f*) data to superpose their high frequency data with that of *ε*″(*f*). The *ε*″(*f*) together with the vertically shifted *M*″(*f*) and *G*″(*f*) are presented in Fig. 1D. It shows, when probed as electric modulus or shear modulus, the maximum of the α-loss peak in *ε*″(*f*) is reduced by about one decade in *M*″(*f*) and *G*″(*f*), while the excess loss/excess wing representing the unresolved JG β-relaxation is unchanged. Hence when KDE is presented by electric modulus *M*″(*f*) or shear modulus *G*″(*f*) formalisms, the α-loss peak is distorted by the presence of by the JG β-relaxation. The shift of the α-loss peaks of *M*″(*f*) and *G*″(*f*) to higher frequencies from that of *ε*″(*f*) by the factor ε_s_/ε_∞_ is slightly larger than one decade. The shift reduces the separation of the α-relaxation from the JG β-relaxation, and it also enhances the merge of the latter with the former. Consequently, the α-loss peaks of *M*″(*f*) and *G*″(*f*) become broader than that found in *ε*″(*f*), and explains why the Kohlrausch exponents ${\beta }_{K}^{M}$ = 0.58 and ${\beta }_{K}^{G}$ = 0.58 are smaller than ${\beta }_{K}^{DS}$ = 0.76 (see Fig. 1A).

#### Phenolphthalein-dimethyl ether (PDE)

**Figure 2:**
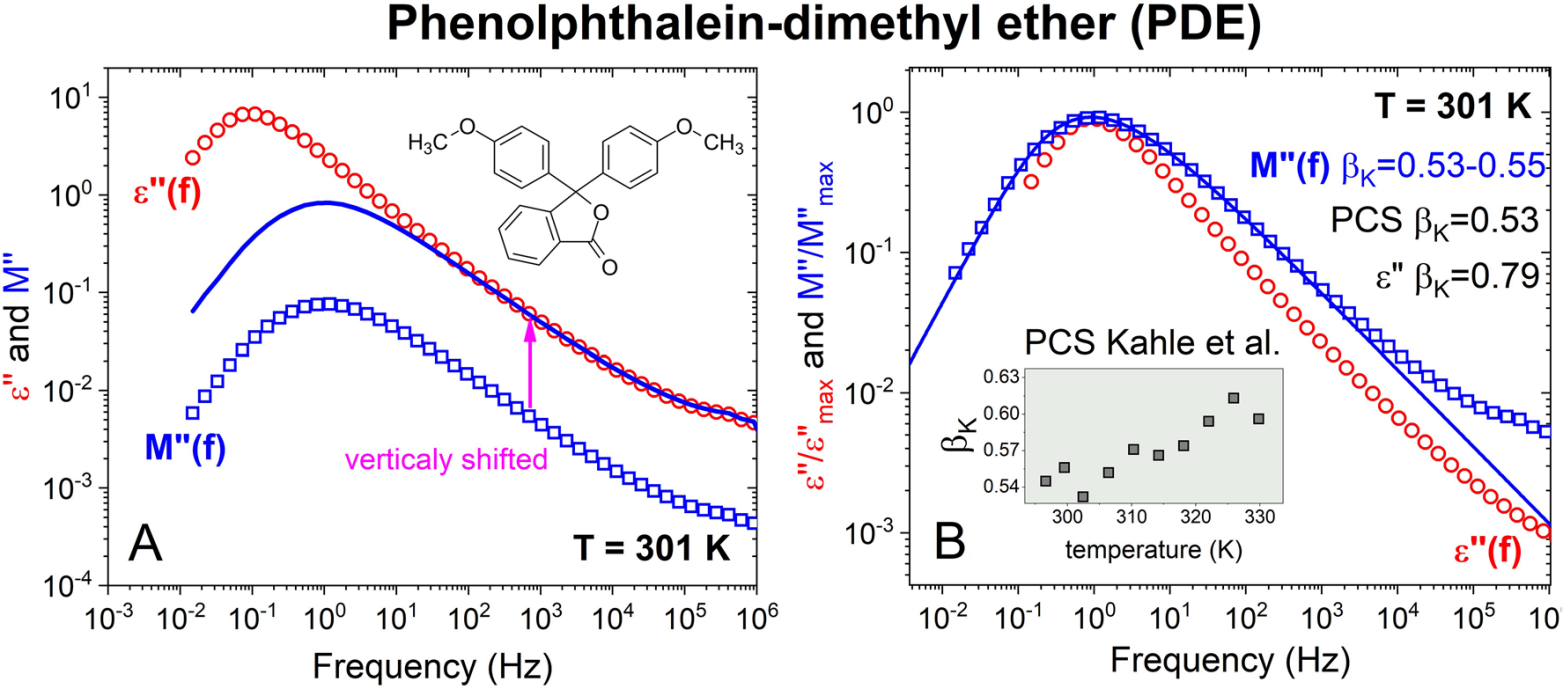
(**A**) Shows data of *ε*″(*f*) and *M*″(*f*) (red and blue scatters, respectively), and the vertically shifted *M*″(*f*) data (solid line) of phenylphthalein-dimethylether (PDE) at *T* = 301 K. In (**B**) data are normalized by the loss maxima and the frequencies to show the broader dispersion in *M*″(*f*) than in *ε*″(*f*) and the ${\beta }_{K}^{M}$ = 0.53–0.55, ${\beta }_{K}^{PCS}$ = 0.53 (see inset from Ref.^39^), and ${\beta }_{K}^{DS}$ = 0.79 at 301 K.

The difference between *ε*″(*f*) and the electric modulus *M*″(*f*) and shear modulus *G*″(*f*) in the frequency dispersion and strength of the α-loss peak of KDE is general for all highly polar glass-formers, and we have more data to show. Figure 2A shows *ε*″(*f*) and *M*″(*f*) data of phenylphthalein-dimethylether (PDE) at *T* = 301 K. *G*″(*f*) data are not available. PDE has Δε = 17.5, similar to Δε = 20 for KDE. In the left panel, the *M*″(*f*) data are shifted vertically to show: (i) the frequency dependence of the excess loss/excess wing representing the JG β-relaxation is the same as in *ε*″(*f*), (ii) there is about one-decade reduction of the intensity of the α-loss peak and (iii) there is about one decade shift to higher frequencies. The similarity in the relation of the shifted *M*″(*f*) to *ε*″(*f*) in PDE and KDE goes together with the comparable values of ${\beta }_{K}^{DS}$, 0.79 for PDE and 0.76 for KDE. The normalized *ε*″(*f*) and *M*″(*f*) data of PDE at *T* = 301 K are compared in Fig. 2B. The Kohlrausch fit of *M*″(*f*) needs a value of ${\beta }_{K}^{M}$ equal to 0.53 or 0.55. PCS measurements of PDE were performed by Kahle et al.^40^. As shown in the inset the Kohlrausch exponent ${\beta }_{K}^{PCS}$ is temperature dependent and the value of 0.51 at 301 K is close to ${\beta }_{K}^{M}$ of *M*″(*f*). This suggests that the cause of the broader dispersion of the α-relaxation seen by PCS than by dielectric spectroscopy is the same as *M*″(*f*).

#### Polychlorinated biphenyl (Aroclor1242)

**Figure 3:**
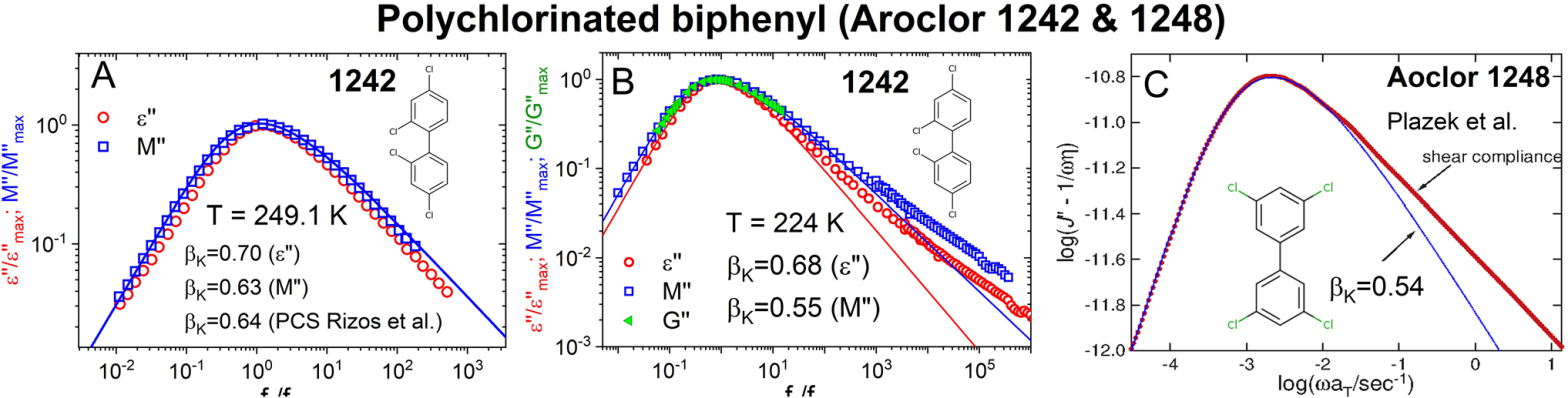
(**A**) Shows the normalized *ε*″(*f*) and *M*″(*f*) vs. normalized frequency of Aroclor 1242 at a higher temperature of 249.1 K. The exponents ${\beta }_{K}^{DS}$ = 0.70 and ${\beta }_{K}^{M}$ = 0.63 from the Kohlrausch fits are compare with ${\beta }_{K}^{PCS}$ = 0.64 from PCS data from Rizos et al.^43^ at about the same temperature. (**B**) Compares the frequency dispersions of the normalized *ε*″(*f*), *M*″(*f*) and *G*″(*f*) at a lower temperature of 224 K. The exponents from the Kohlrausch fits are ${\beta }_{K}^{DS}$ = 0.68 for *ε*″(*f*), and ${\beta }_{K}^{M}$ = 0.55 = ${\beta }_{K}^{G}$ the same for *M*″(*f*) and *G*″(*f*). (**C**) Shows the imaginary part of the complex dynamic compliance (red line), $J"-1/\omega \eta$, fitted by the Fourier transform of the Kohlrausch function with ${\beta }_{K}^{J}$ = 0.54 (blue line)^42^. There is good agreement of ${\beta }_{K}^{J}$ = 0.54 with ${\beta }_{K}^{G}$ and ${\beta }_{K}^{M}$ = 0.53.

Polychlorinated biphenyls also known as Aroclor is another highly polar glass-former having a narrow dielectric loss peak. The dielectric loss spectra of Aroclor 1242^41^ are shown in Fig. 3B at 224 K near *T*_*g*_ = 221 K and at a higher temperature of 249.1 K. The fits by the Kohlrausch functions yield ${\beta }_{K}^{DS}$ = 0.68 at 224 K, and 0.70 at 249.1 K. The corresponding electric modulus *M*″(*f*) loss peak becomes broader as shown by comparing with *ε*″(*f*) after normalizing by the maxima and compensating the shift of *M*″(*f*) to higher frequencies. The values of ${\beta }_{K}^{M}$ for *M*″(*f*) is 0.55 at 224 K, and 0.63 at 249.1 K (see Fig. 3A). Newly acquired shear modulus data of *G*″(*f*) at 224 K are included in the lower panel to show the α-frequency dispersion is the same as *M*″(*f*) and the Kohlrausch exponents ${\beta }_{K}^{G}$ and ${\beta }_{K}^{M}$ are equal to 0.55. Thus the broadening seen in *G*″(*f*) is explained by the decrease of the relaxation strength of the α-relaxation relative to the JG β-relaxation. Plazek et al.^42^ made shear recovery compliance *J*_*r*_(*t*) measurements of Aroclor 1248 having a slightly higher molecular weight than Aroclor 1242 and *T*_*g*_ two degrees higher. From the measurements, the complex dynamic compliance was computed. The imaginary part, $J"-1/\omega \eta$, shown in Fig. 3C is fitted by the Fourier transform of the Kohlrausch function with ${\beta }_{K}^{J}$ = 0.54. The agreement of ${\beta }_{K}^{J}$ = 0.54 with ${\beta }_{K}^{G}$ and ${\beta }_{K}^{M}$ = 0.53 indicates that mechanical spectroscopies (employing either the modulus or compliance modes) broaden the α-relaxation in the same way.

PCS was performed by Rizos et al.^43^ on Aroclor 1242 at temperatures near 249.1 K and higher but not at lower temperatures. The value of ${\beta }_{K}^{PCS}$ reported is 0.64 and temperature independent, which is practically the same as 0.63 for ${\beta }_{K}^{M}$ and we made this clear in Fig. 3. The agreement between ${\beta }_{K}^{PCS}$ and ${\beta }_{K}^{M}$ is like that found in Fig. 2 for PDE. It indicates that the broadening of the α-relaxation seen in dielectric permittivity when probed by PCS is due to a decrease of the relaxation strength of the α-relaxation relative to the unresolved JG β-relaxation close by, enabling the latter to broaden the frequency dispersion of the former.

#### Tributyl phosphate (TBP)

**Figure 4:**
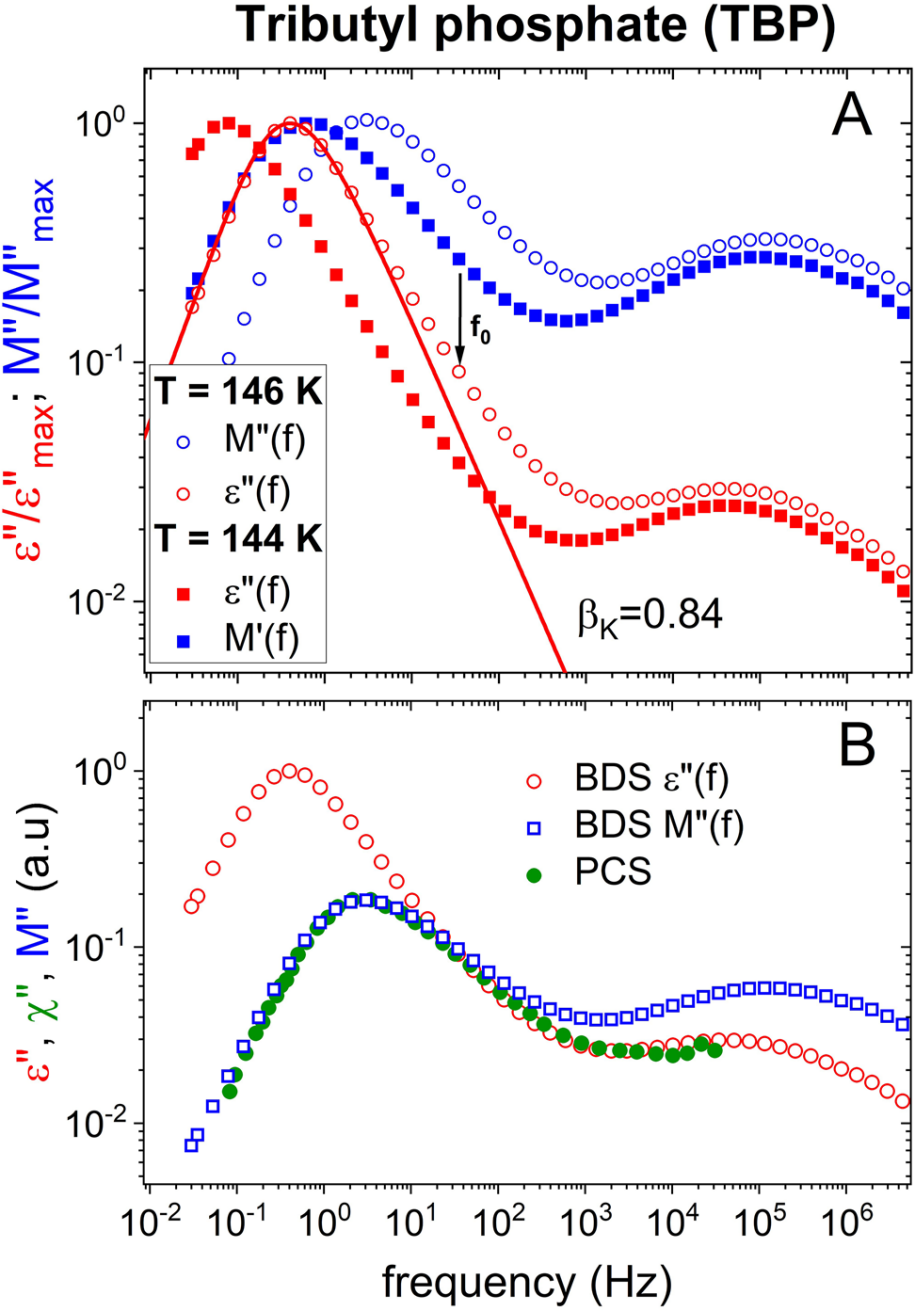
(**A**) Shows the normalized *ε*″(*f*) and *M*″(*f*) vs. frequency at two temperatures 146 and 144 K, and the Kohlrausch fit of *ε*″(*f*) at 146 K with ${\beta }_{K}^{DS}$ = 0.84. The arrow indicates the location of the dielectric primitive relaxation frequency *f*_0_ ≈ *f*_*JG*_ calculated by Eq. (2) with ${\beta }_{K}^{DS}$ = 0.84. (**B**) Shows excellent agreement in frequency dependence between the normalized *M*″(*f*) and χ″(*f*) from PCS (or DLS) obtained by Pabst et al.^21^, which has ${\beta }_{K}^{PCS}$ = 0.49.

The family tributyl phosphate (TBP), triethyl phosphate (TEP), and triphenyl phosphate (TPP) are highly polar glass-formers. TBP has Δε = 20 and narrow dielectric α-loss peak as shown in the upper panel of Fig. 4 and the Kohlrausch fit requires a large ${\beta }_{K}^{DS}$ = 0.84 and similar values for the other members (shown in Fig. S1 for TEP and Fig. S2 for TPP)^21,22,44,45^. This property is like KDE, PDE, aroclor, and glycerol, as well as the other highly polar glass-formers^16,17^ conforming to the correlation of ${\beta }_{K}^{DS}$ with Δε found^15^. However unlike KDE, PDE, and glycerol, TBP and the other members have a prominent dielectric secondary γ-relaxation but it is not the JG β-relaxation, which is unresolved as suggested by the location of the primitive frequency *f*_0_ at 146 K indicated by the arrow in the figure. This difference of TBP and other examples such as diethyl phthalate^36^, dibutyl phthalate^46^, and higher members. TBP and the others do not fall into the class of the so called “type A glass formers”, defined as liquids with dielectric spectra that do not display a discernible secondary relaxation peak (β-relaxation) at temperatures above *T*_*g*_^47^. Nevertheless, these polar glass-formers have larger ${\beta }_{K}^{DS}$ and correlate with Δε as well.

The comparison of the frequency dispersion of the α-loss peak from *ε*″(*f*) and *M*″(*f*) shows the reduction of the relaxation strength of the α-relaxation relative to the excess loss representing the unresolved JG β-relaxation as well as the resolved non-JG γ-relaxation. This is the cause for the broader α-loss peak in *M*″(*f*). In the lower panel of Fig. 4 we compare *M*″(*f*) with the susceptibility χ″(*f*) from PCS (or DLS) obtained by Pabst et al., which has ${\beta }_{K}^{PCS}$ = 0.49^21,22^. There is excellent agreement in frequency dependence between *M*″(*f*) and χ″(*f*).

#### Tripropylene glycol (TPG), methyl tetrahydrofuran (MTHF), diglycyl ether of bisphenol (DGEBA), tricresyl phosphate (TCP), α-phenyl o-cresol

**Figure 5:**
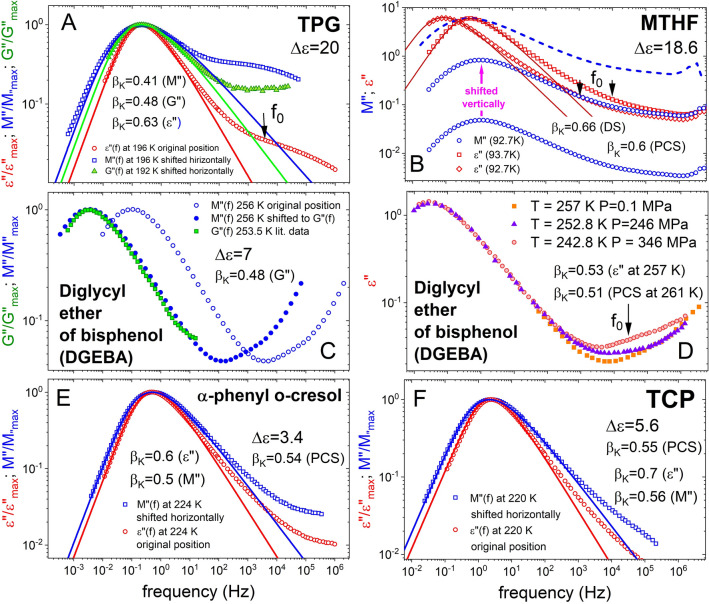
(**A**) Shows the α-loss peak in normalized *ε*″(*f*) is narrower than in the normalized *M*″(*f*), and *G*″(*f*) of TPG, corresponding to the larger value of ${\beta }_{K}^{DS}$ = 0.63 than ${\beta }_{K}^{M}$ = 0.41 and ${\beta }_{K}^{G}$ = 0.48. Data are taken from Ref.^48^. (**B**) Shows the normalized *ε*″(*f*) data vs. frequency at 92.7 and 93.7 K and the *M*″(*f*) data at 92.7 K together with that shifted vertically and the Kohlrausch fit of *ε*″(*f*) with ${\beta }_{K}^{DS}$ = 0.66. The arrow indicates the location of the dielectric primitive relaxation frequency *f*_0_ ≈ *f*_*JG*_ calculated by Eq. (2) with ${\beta }_{K}^{DS}$ = 0.66. The PCS has ${\beta }_{K}^{PCS}$ = 0.60 and NMR has ${\beta }_{K}^{NMR}$ = 0.57^35^. (**C**) Shows the normalized *M*″(*f*) at two temperatures, and normalized *G*″(*f*) at one temperature of DGEBA. The frequency dispersion of *M*″(*f*) and *G*″(*f*) is the same and have the same value of 0.48 for both ${\beta }_{K}^{M}$ and ${\beta }_{K}^{G}$. (**D**) Shows the DGEBA *ε*″(*f*) data vs. frequency at three different combinations of *P* and *T* but the same loss peak frequency. The invariance of the frequency dispersion of the α-relaxation and the K-exponent ${\beta }_{K}^{DS}$ = 0.53 is demonstrated. The arrow indicates the dielectric primitive relaxation frequency *f*_0_ ≈ *f*_*JG*_ calculated by Eq. (2) with ${\beta }_{K}^{DS}$ = 0.53. (**E**) Shows the α-loss peak of α-phenyl o-cresol in normalized *ε*″(*f*) is narrower than in the normalized *M*″(*f*), corresponding to the larger value of ${\beta }_{K}^{DS}$ = 0.60 than ${\beta }_{K}^{M}$ = 0.50, and ${\beta }_{K}^{PCS}$ = 0.54^19^. (**F**) Shows the α-loss peak of tricresyl phosphate (TCP) in normalized *ε*″(*f*) is narrower than in the normalized *M*″(*f*), corresponding to the larger value of ${\beta }_{K}^{DS}$ = 0.70 than ${\beta }_{K}^{M}$ = 0.56, and ${\beta }_{K}^{PCS}$ = 0.55^19^.

These polar glass-formers except TPG all have unresolved JG β-relaxation, and some like MTHF has a fast γ-relaxation. The values of Δε for TPG, MTHF, DGEBA, TCP, and α-phenyl o-cresol in decreasing order are 20, 18.6, 7, 5.6, and 3.4, respectively. These glass-formers are chosen because in addition to *ε*″(*f*) and *M*″(*f*) data either *G*″(*f*) or PCS data are available to compare with. The data shown in Panels A–F of Fig. 5 for TPG^48^, MTHF^34^, DGEBA^49^, TCP^50^, and α-phenyl o-cresol^50^. The smaller value ${\beta }_{K}^{M}$ = 0.41 than ${\beta }_{K}^{G}$ = 0.48 of TPG is explained by lesser sensitivity of shear modulus than electric modulus. The PCS data of MTHF, TCP, and α-phenyl o-cresol are not reproduced from the publications except their respective ${\beta }_{K}^{PCS}$ values of 0.60, 0.51, 0.55, and 0.54. The approximate agreement of ${\beta }_{K}^{PCS}$ = 0.55 with ${\beta }_{K}^{M}$ = 0.56 in TCP as well as ${\beta }_{K}^{PCS}$ = 0.54 with ${\beta }_{K}^{M}$ = 0.50 in α-phenyl o-cresol is worth notice for supporting the explanation given.

In the case of DGEBA, Panel C of Fig. 5 show good agreement in the frequency dispersion of the α-loss peak between *G*″(*f*) at 253.5 K and *M*″(*f*) at 256 K with both having the same Kohlrausch exponent, ${\beta }_{K}^{G}$ = 0.46 = ${\beta }_{K}^{M}$. At 261 K, the lowest temperature of the PCS experiment^51^, the value of ${\beta }_{K}^{PCS}$ is 0.51, while it is 0.55 at 263 K. The temperature dependence of ${\beta }_{K}^{PCS}$ makes uncertain its value at 253.5 K, ten degrees lower, to compare with ${\beta }_{K}^{G}$(253.5 K) = 0.46. On the other hand, the α-loss peak in *ε*″(*f*) at 257 K is narrower with a larger ${\beta }_{K}^{DS}$ = 0.53^52^. The arrow in Panel D of Fig. 5 indicates the dielectric primitive relaxation frequency *f*_0_ ≈ *f*_*JG*_ calculated by Eq. (2) with ${\beta }_{K}^{DS}$ = 0.53. The fact that *f*_0_ is much higher than the α-loss peak frequency suggests the broadening in going from *ε*″(*f*) to *M*″(*f*) or *G*″(*f*) and χ″(*f*) from PCS is not large. This is consistent with the small difference between ${\beta }_{K}^{PCS}$ = 0.51 and ${\beta }_{K}^{DS}$ = 0.53.

#### New ε″(f), M″(f), and G″(f) experimental data of highly polar glass-formers

**Figure 6:**
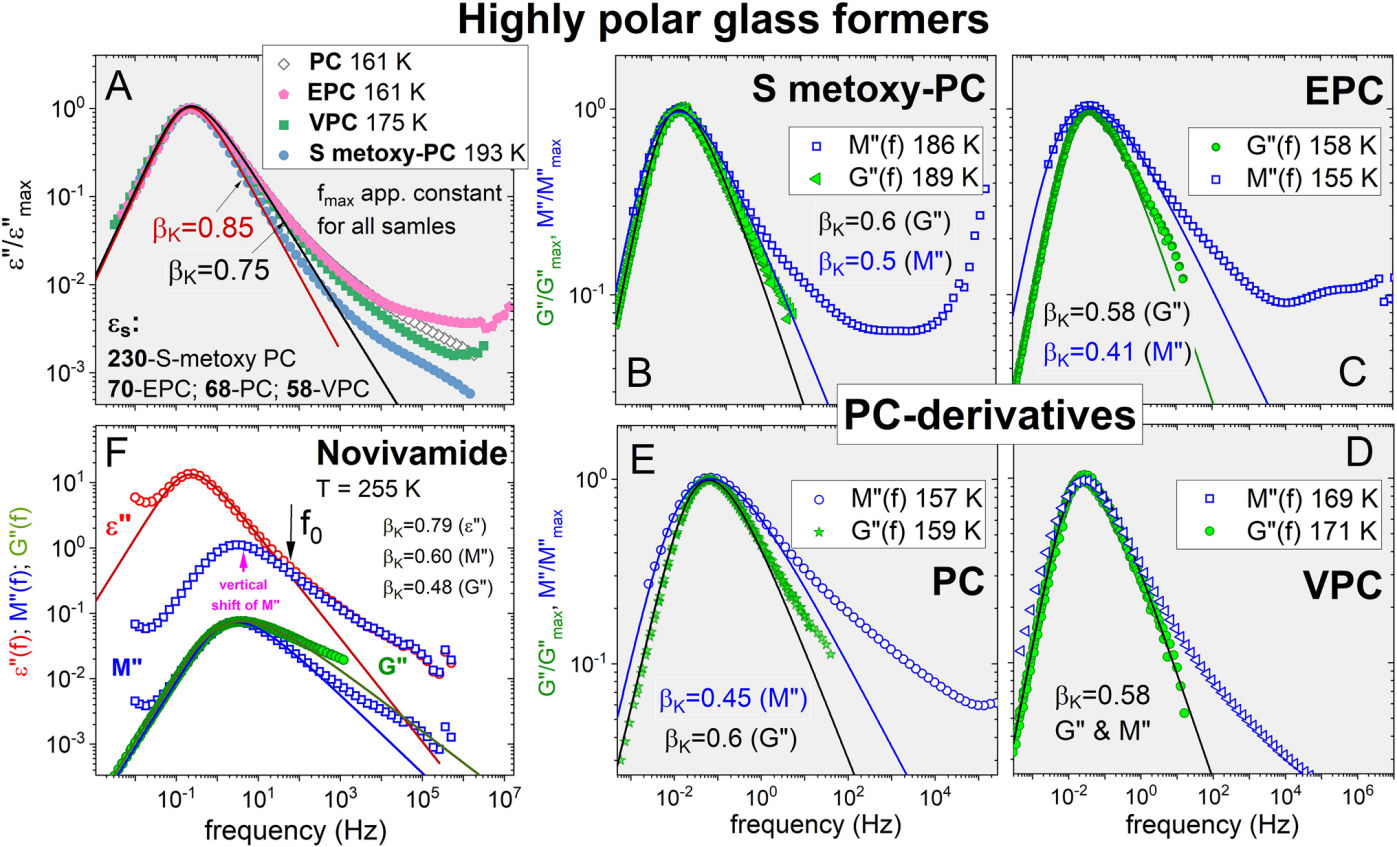
(**A**) Shows the narrow dielectric α-loss peaks of propylene carbonate (PC) and the PC derivatives, S metoxy-PC, EPC, and VPC. The Kohlrausch fits have ${\beta }_{K}^{DS}$ falling within the range from 0.75 to 0.85 and correlate with Δε. (**B**) Shows the normalized *M*″(*f*) at 186 K, and the normalized *G*″(*f*) at 189 K of S metoxy-PC, and the Kohlrausch fits with ${\beta }_{K}^{M}$ = 0.50 and ${\beta }_{K}^{G}$ = 0.60. (**C**) Shows the normalized *M*″(*f*) at 155 K, and the normalized *G*″(*f*) at 158 K of EPC, and the Kohlrausch fits with ${\beta }_{K}^{M}$ = 0.41 and ${\beta }_{K}^{G}$ = 0.58. (**D**) Shows the normalized *M*″(*f*) at 169 K, and the normalized *G*″(*f*) at 171 K of VPC, and the Kohlrausch fits with ${\beta }_{K}^{M}$ = 0.58 and ${\beta }_{K}^{G}$ = 0.58. (**E**) Shows the normalized *M*″(*f*) at 186 K, and the normalized *G*″(*f*) at 189 K of PC, and the Kohlrausch fits with ${\beta }_{K}^{M}$ = 0.45 and ${\beta }_{K}^{G}$ = 0.60. (**F**) Shows data of *ε*″(*f*) and *M*″(*f*), and the vertically shifted *M*″(*f*) data of the pharmaceutical novivamide at *T* = 255 K, and the Kohlrausch fits with ${\beta }_{K}^{DS}$ = 0.79, ${\beta }_{K}^{M}$ = 0.60 and ${\beta }_{K}^{G}$ = 0.48.

To bolster the experimental support of the explanation, we made new measurements of *G*″(*f*) over the range, 10^–2^ < *f* < 20 Hz, of several highly polar glass-formers for which *ε*″(*f*) were also measured and represented together in Fig. 6. These include propylene carbonate (PC) and its three derivatives: S-methoxy PC, 4-vinyl-1,3-dioxolan-2-one (VPC) and 4-ethyl-1,3-dioxolan-2-one (EPC). The dielectric experimets of PC-derivatives in a frequency range from 10^–3^ Hz to 10^7^ Hz were carried out by means of dielectric spectrometer (alpha Novo-Control GMBH with novocool system). The stainless steel capacitor (diameter = 15 mm; distance of 0.098 mm provided by quartz) was used for measurements. ARES G2 Rheometer was used to determine the mechanical properties of PC-derivatives. The shear modulus measurements were performed by means of aluminum parallel plates of diameter = 4 mm.

The width of the dielectric α-relaxation in S-methoxy PC is narrowest and its value of 0.85 for ${\beta }_{K}^{DS}$ at *T*_*g*_ is the largest recorded for highly polar glass-formers consistent with its large Δε = 230. Dielectric loss peaks of the other three systems are slightly broader with ${\beta }_{K}^{DS}$ equals to 0.75 and smaller Δε in the range from 58 to 70. The corresponding *M*″(*f*) calculated from *ε*″(*f*) are compared with *G*″(*f*) for all four glass-formers in Fig. 6. Again the α-relaxation frequency dispersion of *M*″(*f*) and *G*″(*f*) are broader than that of *ε*″(*f*), and the values of ${\beta }_{K}^{M}$ and ${\beta }_{K}^{G}$ are smaller. More important is the reduction in the relaxation strength of the α-loss peak relative to the excess loss representing the unresolved JG β-relaxation when represented by *M*″(*f*) or measured in terms of *G*″(*f*). The *ε*″(*f*) changes by more than two decades from the α-loss peak to the excess wing. By contrast, the corresponding change in *M*″(*f*) and *G*″(*f*) is about one decade. Again the difference means that the α-relaxation seen by dielectric relaxation and characterized by the larger ${\beta }_{K}^{DS}$ is real because it is not modified by the much weaker JG β-relaxation despite the latter is close by. On the other hand, the broader modulus peaks characterized by smaller ${\beta }_{K}^{M}$ and ${\beta }_{K}^{G}$ is unreal because of the reduction in the disparity between the relaxation strengths of the two processes.

We made dielectric *ε*″(*f*) and shear modulus *G*″(*f*) measurements of novivamide with a chemical structure different from PC and PC derivatives. The comparison of *M*″(*f*) and *G*″(*f*) with *ε*″(*f*) in Fig. 6F supports once more the explanation of the difference given before for the other polar glass-formers.

### Weakly polar glass-formers

According to Eq. (3) the separation of the JG β-relaxation from the α-relaxation is proportional to (1 − ${\beta }_{K}^{DS}$), the test is best carried out in weakly polar glass-formers with smaller ${\beta }_{K}^{DS}$ or wider dielectric α-loss peak. Since the JG β-relaxation is the slowest among secondary relaxations, the condition guarantees the non-JG γ-relaxation if present will be further away from the α-relaxation. With the JG β-relaxation far away from the α-relaxation, the change in the representation of dielectric data from *ε*″(*f*) to *M*″(*f*) will not alter the frequency dispersion of the α-loss peak. This is because the JG β-relaxation has either no or minimal effect on the frequency dispersion of the α-loss peak on changing from *ε*″(*f*) to *M*″(*f*), by contrast with polar glass-formers. The *G*″(*f*) from shear modulus measurement and *χ*″(*f*) from PCS are expected to have approximately the same frequency dispersion as *ε*″(*f*) and *M*″(*f*). We carried out the test of the expected behavior of weakly polar glass-formers by analyzing dielectric, shear modulus, and PCS data of some weakly polar glass-formers. The results are reported in the subsections to follow. Körber et al.^19^ had already shown the dielectric ${\beta }_{K}^{DS}$ is comparable in value to either the PCS ${\beta }_{K}^{PCS}$ or the NMR ${\beta }_{K}^{NMR}$ in the better known non-polar glass-formers, including OTP, trinaphthal benzene, and toluene. All these three weakly polar glass-formers have smaller ${\beta }_{K}^{DS}$ of about 0.50 and the JG β-relaxation is widely separated from the α-relaxation. Hence it does not broaden the α-relaxation when probed by PCS and NMR.

#### 1,1′-bis (p-methoxyphenyl) cyclohexane (BMPC)

**Figure 7:**
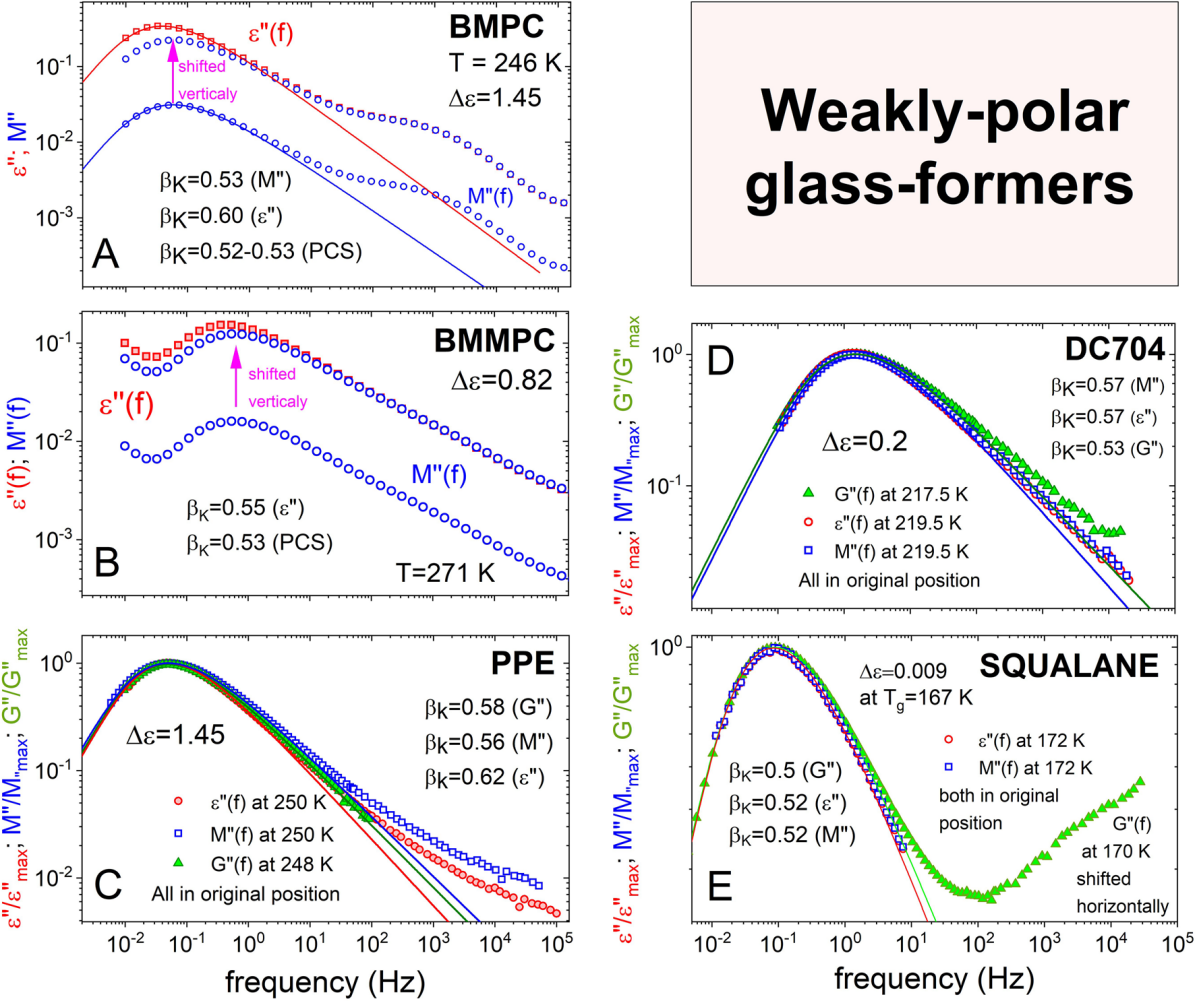
(**A**) Shows the *ε*″(*f*) and *M*″(*f*) data of BMPC at 246 K, and the vertically shifted *M*″(*f*). The K-exponents are ${\beta }_{K}^{DS}$ = 0.60, ${\beta }_{K}^{M}$ = 0.53, and ${\beta }_{K}^{PCS}$ = 0.52–0.53 for the α-relaxation in *ε*″(*f*), *M*″(*f*), and PCS respectively. (**B**) Shows the data of BMMPC. Similar to (**A**), and ${\beta }_{K}^{DS}$ = 0.55, ${\beta }_{K}^{M}$ ≈ 0.55, and ${\beta }_{K}^{PCS}$ = 0.53. (**C**) Shows the normalized *ε*″(*f*), *M*″(*f*), and *G*″(*f*) data of PPE at 250 K and 248 K, and the Kohlrausch fits with ${\beta }_{K}^{DS}$ = 0.62, ${\beta }_{K}^{M}$ ≈ 0.56, and ${\beta }_{K}^{G}$ = 0.58. (**D**) shows the normalized *ε*″(*f*), *M*″(*f*), and *G*″(*f*) data of DC704 at 219.5 K and 217.5 K, and the Kohlrausch fits with ${\beta }_{K}^{DS}$ = 0.57, ${\beta }_{K}^{M}$ = 0.57, and ${\beta }_{K}^{G}$ = 0.53. (**E**) shows the normalized *ε*″(*f*), *M*″(*f*), and *G*″(*f*) data of squalane at 172 K and 170 K, and the Kohlrausch fits with ${\beta }_{K}^{DS}$ = 0.52, ${\beta }_{K}^{M}$ = 0.52, and ${\beta }_{K}^{G}$ = 0.50.

BMPC was also known before as bis-phenol-C-dimethylether (BCDE) has Δε = 1.45 not as low as the ideal non-polar glass-formers like OTP, tri-naphthyl benzene (TNB), and toluene. The *ε*″(*f*) data were taken from Hensel-Bielowka et al.^53^, and not from the earlier work by Meier et al.^54^ This is because in the later work, the Kohlrausch fit to the data was done in the same way as the other glass-formers in this paper, giving the value of ${\beta }_{K}^{DS}$ = 0.60. On the other hand, the fit in the earlier work was not shown and a smaller value of 0.51 was reported and used by Körber et al.^19^. The calculated *M*″(*f*) at 246 K is presented in Fig. 7A. The *M*″(*f*) data are shifted vertically to have the excess loss in *M*″(*f*) coalescing with that in *ε*″(*f*) like done before for KDE in Fig. 1D. The difference in the height of the α-loss peak between the shifted *M*″(*f*) and *ε*″(*f*) is a factor of 1.5 compared to 10 in the case of KDE. The width of the α-relaxation in *M*″(*f*) is still larger than in *ε*″(*f*) as reflected by ${\beta }_{K}^{M}$ = 0.53 compared to ${\beta }_{K}^{DS}$ = 0.60^53^. The PCS data we consider are not from Meier et al. but from a later work published by the same group in Mainz by Patkowski et al.^55^, again because the more accurate data and analysis performed. The value of ${\beta }_{K}^{PCS}$ for PCS decreases on lowering temperature and by extrapolation of the trend its value at *T*_*g*_ = 247 K is estimated to be 0.52–0.53^55^. Hence there is good agreement between ${\beta }_{K}^{M}$ = 0.53 and ${\beta }_{K}^{PCS}$ = 0.52–0.53 of BMPC, in accord with the prediction.

#### 1,1′-bis(p-methoxyphenyl) cyclohexane (BMMPC)

The glass-former BMMPC, also referred to in the literature as bis-kresol-C-dimethylether (BKDE) is closely related in chemical structure to BMPC and its *T*_*g*_ is 263 K. It has Δε = 0.82 and the value of 0.55 for the dielectric Kohlrausch exponent ${\beta }_{K}^{DS}$ at 264.1 K^56^. The smaller value of ${\beta }_{K}^{DS}$=0.55 implies the JG β-relaxation is widely separated from the α-relaxation. The dielectric loss ε″(*f*) at 271 K is shown in Fig. 7B together with the calculated *M*″(*f*). The vertically shifted *M*″(*f*) remarkably has the same frequency dependence as ε″(*f*) except for the slight horizontal shift due to change from susceptibility to modulus. This feature validates the prediction of no broadening in going from ε″(*f*) to *M*″(*f*) because the JG β-relaxation is well separated from the α-relaxation and has no effect in broadening the α-relaxation in *M*″(*f*). PCS data of BMMPC were published by Patkowski et al.^55^. The PCS Kohlrausch exponent ${\beta }_{K}^{PCS}$ is temperature dependent with values decreasing with falling temperature in the range 0.53–0.62. There is good agreement between ${\beta }_{K}^{DS}$ = 0.55 and ${\beta }_{K}^{PCS}$ = 0.53 at temperatures near *T*_*g*_ = 263 K, and this result provides strong support of the prediction.

#### Polyphenyl ether (PPE)

The value Δε = 1.45 of PPE is nearly the same as BMPC. The frequency dispersions of the α-loss peaks in *ε*″(*f*), *M*″(*f*), and *G*″(*f*) are compared in Fig. 7C. From the Kohlrausch fits, the values of the exponents ${\beta }_{K}^{DS}$, ${\beta }_{K}^{M}$, and ${\beta }_{K}^{G}$ are 0.62, 0.56, and 0.58 respectively. Thus the α-relaxations in *M*″(*f*), and *G*″(*f*) have effectively the same frequency dispersion, and it is slightly narrower in *ε*″(*f*). The situation is similar to BMPC.

#### Tetramethyltetraphenyltrisiloxane (DC704)

The value Δε = 0.2 of DC704 is an order of magnitude smaller than BMPC and PPE, and should be a better candidate to test the expected behavior. The frequency dispersions of the α-loss peaks in *ε*″(*f*), *M*″(*f*), and *G*″(*f*) from Ref.^57^ are compared in Fig. 7D. The frequency dispersions of *ε*″(*f*) and *M*″(*f*) are identical, and the exponents ${\beta }_{K}^{DS}$ and ${\beta }_{K}^{M}$ are equal to 0.57. The equality is testament to the prediction of no change in the frequency dispersion in going from *ε*″(*f*) to *M*″(*f*) when the JG β-relaxation is far away from the α-relaxation. Moreover, the frequency dispersion in *G*″(*f*) is only slightly broader with the exponent ${\beta }_{K}^{G}$ = 0.53, a bit lower than 0.57 of ${\beta }_{K}^{DS}$ and ${\beta }_{K}^{M}$. By the way, the α-loss peak in *χ*″(*f*) from PCS measurements on DC704 has the same frequency dispersion as in *ε*″(*f*) and *G*″(*f*)^58^. Thus we have overwhelming evidence from DC704 to validate the predicted difference in the behavior of non-polar glass-formers than polar glass-formers when probed by methods different from dielectric susceptibility.

#### Perhydrosqualene (squalane)

Squalane has the smallest Δε among the examples given. The value is 0.009 according to Richert et al.^59^ and 0.015 from Jakobsen et al.^48^. The frequency dispersions of the α-loss peaks in *ε*″(*f*) and *G*″(*f*) from Ref.^48^ are compared in Fig. 7E together with *M*″(*f*) we calculated from ε*(*f*). On first look, it seems the α-loss peaks in *ε*″(*f*), *M*″(*f*), and *G*″(*f*) are all the same. Indeed the frequency dispersions of *ε*″(*f*) and *M*″(*f*) are identical, and the exponents ${\beta }_{K}^{DS}$ and ${\beta }_{K}^{M}$ are equal to 0.52. The one in *G*″(*f*) is almost the same with ${\beta }_{K}^{G}$ = 0.50. Like DC704 and polybutadiene, the data of squalene fully verify the prediction that the frequency dispersion of weakly polar glass-formers with smaller dielectric ${\beta }_{K}^{DS}$ is not broadened when probed by shear modulus or PCS.

#### Polybutadiene

**Figure 8:**
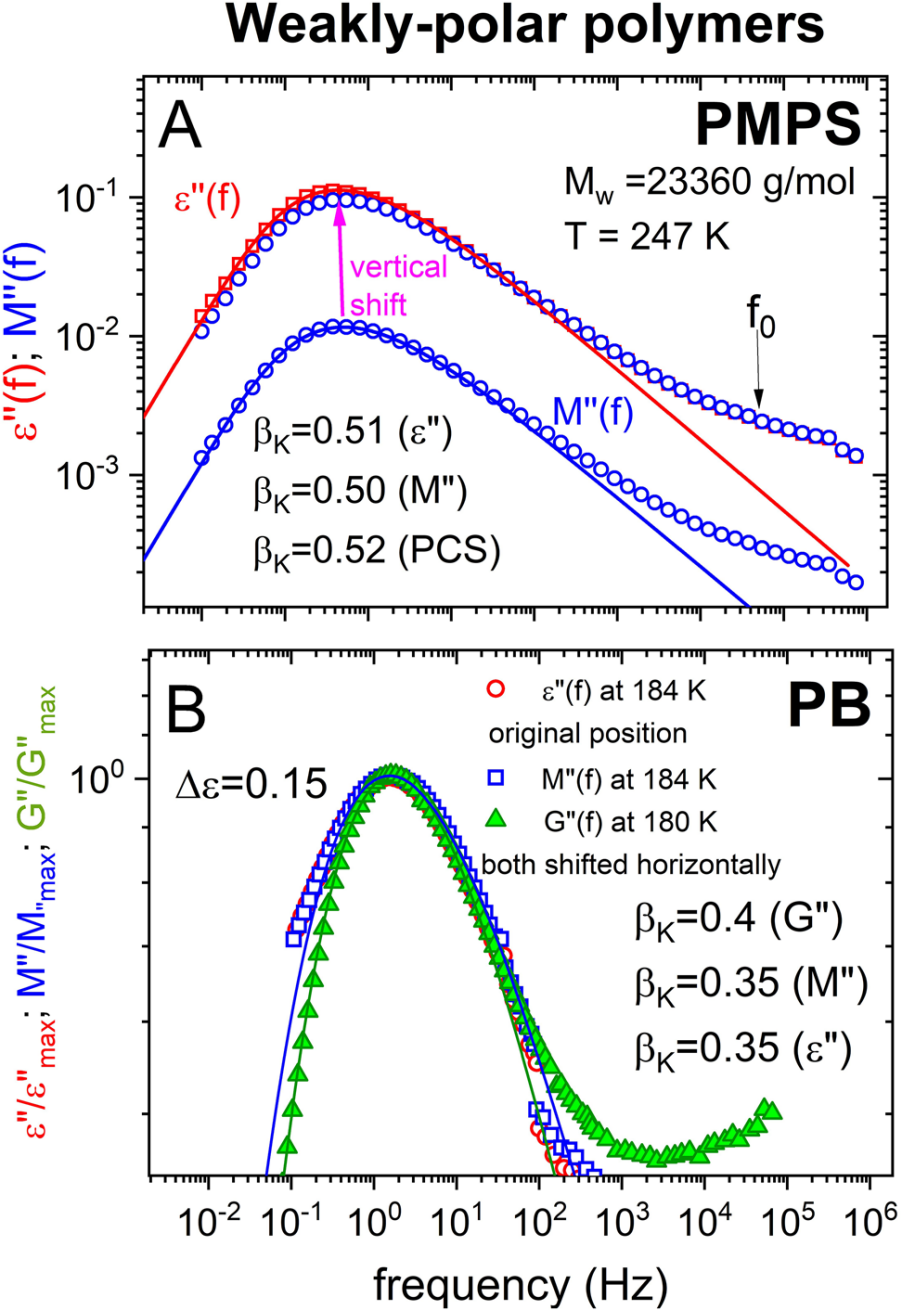
(**A**) Shows the *ε*″(*f*) and *M*″(*f*) data of the polymer PMPS at 247 K, and the vertically shifted *M*″(*f*). The K-exponents are ${\beta }_{K}^{DS}$ = 0.51, ${\beta }_{K}^{M}$ = 0.50, and ${\beta }_{K}^{PCS}$ = 0.52 for the α-relaxation in *ε*″(*f*), *M*″(*f*), and PCS respectively. The arrow indicates the dielectric primitive relaxation frequency *f*_0_ ≈ *f*_*JG*_ calculated by Eq. (2) with ${\beta }_{K}^{DS}$ = 0.51. (**B**) Shows the normalized *ε*″(*f*), *M*″(*f*), and *G*″(*f*) data of the polymer polybutadiene at 184 K and 180 K, and the Kohlrausch fits with ${\beta }_{K}^{DS}$ = 0.35, ${\beta }_{K}^{M}$ = 0.35, and ${\beta }_{K}^{G}$ = 0.40.

The polymer polybutadiene (PB) with a molecular weight of 5000 g/mol has Δε = 0.15 was studied by dielectric and shear modulus^48^. The frequency dispersion of the α-loss peaks in *ε*″(*f*) and *G*″(*f*) are shown in Fig. 8B together with *M*″(*f*) we calculated from ε*(*f*). The frequency dispersions of *ε*″(*f*) and *M*″(*f*) are identical, and the exponents ${\beta }_{K}^{DS}$ and ${\beta }_{K}^{M}$ are equal to 0.35. Such a small value of ${\beta }_{K}^{DS}$ leads to JG β-relaxation widely separated from the α-relaxation, and is ideal for testing the prediction. It follows that the JG β-relaxation has no effect in changing the frequency dispersion of the α-relaxation in going from *ε*″(*f*) to *M*″(*f*). Moreover, the frequency dispersion in *G*″(*f*) is only slightly narrower with the exponent ${\beta }_{K}^{G}$ = 0.40, slightly larger than 0.35 of ${\beta }_{K}^{DS}$ and ${\beta }_{K}^{M}$. Like DC704, the data of polybutadiene provide strong support for the prediction for weakly polar glass-formers.

#### Poly(methylphenylsiloxane) (PMPS)

PMPS is another weakly polar polymer. Dielectric permittivity measurements on a sample with a molecular weight of 23,360 g/mol were made by Paluch et al.^60^. PCS measurements on a slightly higher molecular weight of 28,500 g/mol were made by Boese et al.^61^. The *ε*″(*f*) data at 247 K and the calculated *M*″(*f*) are shown in Fig. 8A together with the vertically shifted *M*″(*f*). The frequency dispersions of the α-relaxation in *ε*″(*f*) and *M*″(*f*) are almost the same as evidenced by the Kohlrausch fit to *ε*″(*f*) and *M*″(*f*) with exponents ${\beta }_{K}^{DS}$ = 0.51 and ${\beta }_{K}^{M}$ = 0.50 respectively. The maximum of the shifted *M*″(*f*) is reduced from that of *ε*″(*f*) by a small factor of 0.88. The PCS Kohlrausch exponent ${\beta }_{K}^{PCS}$ reported by Boese et al. at the lowest temperature of 253 K has the value of 0.52, close to ${\beta }_{K}^{DS}$ = 0.51 and ${\beta }_{K}^{M}$ = 0.50 from *ε*″(*f*) and *M*″(*f*). Hence the frequency dispersion of the α-relaxation is practically unchanged in *ε*″(*f*), *M*″(*f*), and PCS in PMPS. This is consistent with our prediction since the Kohlrausch exponents are smaller and the JG β-relaxation is widely separated from the α-relaxation as suggested by the location of the primitive relaxation frequency *f*_0_ in Fig. 8A.

## Discussion and conclusion

**Figure 9:**
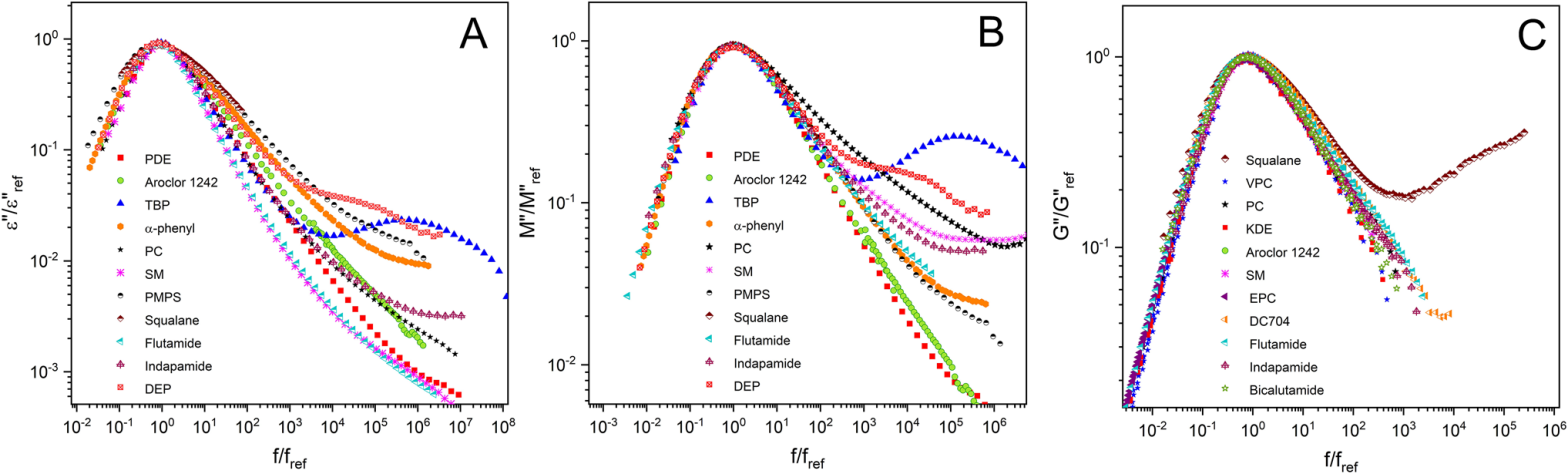
Dielectric (**A,B**) and mechanical (**C**) data of polar and weekly polar glass-formers. The data of PC, flutamide, indapamide, DEP, bicalutamide, EPC and VPC were obtained in this work.

A serious challenge to a verity of the dynamics of polar glass-formers obtained by dielectric permittivity spectroscopy (DS) was issued by the recent publications by Körber et al.^19^, Gabriel et al.^62^, and Pabst et al.^22^. The challenge is that the narrow frequency dispersion of the intense dielectric α-loss peak in *ε*″(*f*) becomes much broader when the same material is probed by other spectroscopies including PCS (depolarized light scattering) and NMR^19,36^ and also shear mechanical modulus *G*″(*f*)^21^. The exponent ${\beta }_{K}^{DS}$ of the Kohlrausch fit to the dielectric loss peak in *ε*″(*f*) is significantly larger than the exponents, ${\beta }_{K}^{PCS}$, ${\beta }_{K}^{NMR}$, and ${\beta }_{K}^{G}$. We confirmed this discrepancy in several polar glass-formers from the literature as well as by making our own dielectric ε*(*f*) and G*(*f*) measurements on additional compounds (see Fig. 9). On the other hand, the relaxation times ${\tau }_{\alpha }^{DS}$ of DS, though different from ${\tau }_{\alpha }^{PCS}$ and ${\tau }_{\alpha }^{G}$ due to the difference in correlation functions, all have similar temperature dependence. This discrepancy, however, does not occur in weekly polar glass-formers where the width of ε*(*f*) function is approximately the same as those of the others techniques.

As it stands, the general finding casts serious doubt on the verity of the narrow frequency dispersion and the large value of ${\beta }_{K}^{DS}$ of polar glass-formers taken by dielectric spectroscopy. Potentially. the worth of dielectric spectroscopy in the study of the dynamics of glass-formers, and validity of the results from DS accumulated over the past hundred years, as well as the recently found correlation of ${\beta }_{K}^{DS}$ with Δε found by Paluch et al., are questioned^15^ and repeatedly verified by others^18,19^. The seriousness of the situation requires an in-depth consideration of the dynamics of polar glass-formers, not only the structural α-relaxation but also the presence of the accompanying and universal JG β-relaxation. Empirically the excess loss/excess wing in *ε*″(*f*) data of polar glass-formers indicates the JG β-relaxation is present although unresolved, and it is located nearly the α-relaxation on its high-frequency flank. This property of the JG β-relaxation as seen by DS is consistent with the correlation of log(*τ*_α_/*τ*_JG_) with (1 − *β*_*K*_) given by Eq. (3) from the Coupling Model (CM). The dielectric strength of the unresolved JG β-relaxation is small compared to the α-relaxation. Thus it has no effect on the full-width at half-maximum of the frequency dispersion of the α-relaxation or the value of the exponent ${\beta }_{K}^{DS}$ of the Kohlrausch fit. Thus the larger value of ${\beta }_{K}^{DS}$ from dielectric permittivity truly characterizes the dynamics of the α-relaxation of polar glass-formers.

It is essential to consider not just the change of the α-relaxation alone but also the JG β-relaxation altogether when going from *ε*″(*f*) of dielectric permittivity of polar glass-formers to *G*″(*f*) of shear modulus or *χ*″(*f*) of PCS and NMR. The changes in the relaxation strengths of the two processes are not necessarily uniform. Thus the narrow frequency dispersion of the α-relaxation seen in *ε*″(*f*) can change substantially when probed by the other spectroscopies. We substantiate this possibility by changing the representation of dielectric measurements from *ε*″(*f*) to the electric modulus *M*″(*f*). For the polar glass-formers, we found the narrow frequency dispersion of the α-loss peak in *ε*″(*f*) becomes a broad peak in *M*″(*f*) (see Fig. 9A,B). The cause is traced to the more significant reduction of the dielectric strength of the α-relaxation relative to that of the JG β-relaxation. Since the two relaxations in polar glass-formers are not widely separated already in *ε*″(*f*), the disparity in the changes of their relaxation strengths in conjunction with the additional decrease in the separation of their relaxation time gives rise to the broadening of the α-loss peak in *M*″(*f*). This explanation of broadening of the α-relaxation in *M*″(*f*) applies verbatim to *G*″(*f*) since both are modulus and is supported by *M*″(*f*) and *G*″(*f*) from experiments having either nearly the same frequency dispersion in a number of glass-formers shown in Fig. 9. So is the good agreement of *χ*″(*f*) from PCS with *M*″(*f*), or the Kohlrausch exponents ${\beta }_{K}^{DS}$ and ${\beta }_{K}^{PCS}$ being about the same, in some glass-formers. By explaining the broadening of the dielectric α-loss peak of polar glass-formers when probed by other techniques, we have two crucial conclusions. The narrow width of the dielectric loss peak in *ε*″(*f*) and the associated larger ${\beta }_{K}^{DS}$ truly reflect the heterogeneous and cooperative molecular dynamics of the α-relaxation in polar glass-formers because it is unaffected by the much weaker JG β-relaxation despite it is nearby. Thus there is nothing wrong with dielectric spectroscopy in applying it to study the dynamics of polar glass-formers. By contrast, the broadened ‘α’-relaxation observed by *G*″(*f*) or by PCS and NMR has the α-relaxation admixed with the JG β-relaxation, and its smaller exponents, ${\beta }_{K}^{G}$, ${\beta }_{K}^{PCS}$, and ${\beta }_{K}^{NMR}$ do not characterize the genuine α-relaxation of polar glass-formers. In other words, for polar glass-formers having narrow dielectric α-loss peak and larger ${\beta }_{K}^{DS}$, the broader frequency dispersion of the α-relaxation deduced from shear modulus, PCS, and NMR are not factual. Needless to say, the correlation of ${\beta }_{K}^{DS}$ with Δε found by dielectric spectroscopy and the theoretical rationalization^15^ remain valid.

Our explanation of the effect found in polar glass-formers with narrow α-loss peak implies that the dielectric α-relaxation is not broadened in glass-formers having the JG β-relaxation widely separated from the α-relaxation, whether they are polar or not. According to Eq. (3), the separation, log(*τ*_α_/*τ*_JG_), is proportional to (1 − ${\beta }_{K}^{DS}$). Hence a corollary of the explanation is the absence of a significant change of the frequency dispersion in glass-formers with larger (1 − ${\beta }_{K}^{DS}$) or smaller ${\beta }_{K}^{DS}$. Molecular glass-formers having smaller ${\beta }_{K}^{DS}$ and larger log(*τ*_α_/*τ*_JG_) are usually non-polar like OTP, TNB, and toluene, studied before by DS^47,59,63,64^, PCS^65,66^, shear compliance *J*(*t*)^67^. The dielectric ${\beta }_{K}^{DS}$ of these three glass-formers have values of about 0.51 close to those of ${\beta }_{K}^{PCS}$ and ${\beta }_{K}^{J}$, and thus verifying the prediction directly. We have more non-polar glass-formers with smaller ${\beta }_{K}^{DS}$ in showing first the broad frequency dispersion of *ε*″(*f*) is either unchanged or hardly changed when replaced by *M*″(*f*). Furthermore, ${\beta }_{K}^{DS}$ and ${\beta }_{K}^{M}$ are nearly the same as ${\beta }_{K}^{PCS}$ or ${\beta }_{K}^{G}$, whichever is available. The amount of data confirm the predicted behavior of weakly polar glass-formers with smaller ${\beta }_{K}^{DS}$ to be different from the polar glass-formers with larger ${\beta }_{K}^{DS}$, and strengthens the explanation for the polar glass-formers.

The ubiquitous presence of the JG β-relaxation and the inseparable relations of its relaxation times to that of the α-relaxation (Eq. (3)) are supported by many corroborative evidences such as given in Refs.^34,68–73^ are critical in restoring the verity of the dynamics obtained by using dielectric permittivity spectroscopy of polar glass-formers. On the other hand, the presence of the JG β-relaxation and its relations to the α-relaxation was not recognized in the papers of Körber et al.^19,74^, Gabriel et al.^21,62^, and Pabst et al.^22^, and the consequence is that they were not able to reach the same conclusion.

## Supplementary Information

Supplementary Information.

## References

[1] Kohlrausch R (1854). Theorie des elektrischen Rückstandes in der Leidener Flasche. Ann. Phys. Chem..

[2] Debye, P. *Ber. Deut. Phys. Ges.* Summary in: Polare Molekeln (Polar Molecules), Leipzig, 1929; Handbuck der Radiologie, Vol. VI, Part 2, 2nd ed. **55**, 777 (1913) (1934).

[3] Debye P (1929). Polar Molecules.

[4] Davidson DW, Cole RH (1951). Dielectric relaxation in glycerol, propylene glycol, and n-propanol. J. Chem. Phys..

[5] Kremer F, Schönhals A (2003). Broadband Dielectric Spectroscopy.

[6] Lunkenheimer P, Schneider U, Brand R, Loid A (2000). Glassy dynamics. Contemp. Phys..

[7] Johari GP, Goldstein M (1970). Viscous liquids and the glass transition. II. Secondary relaxations in glasses of rigid molecules. J. Chem. Phys..

[8] Hensel-Bielowka S, Paluch M, Ngai KL (2005). Emergence of the genuine Johari-Goldstein secondary relaxation in m-fluoroaniline after suppression of hydrogen-bond-induced clusters by elevating temperature and pressure. J. Chem. Phys..

[9] Ngai KL, Casalini R, Capaccioli S, Paluch M, Roland CM (2005). Do theories of the glass transition, in which the structural relaxation time does not define the dispersion of the structural relaxation, need revision?. J. Phys. Chem. B.

[10] Ngai KL (2005). Do theories of glass transition that address only the α-relaxation need a new paradigm?. J. Non-Cryst. Solids.

[11] Ngai KL, Paluch M (2004). Classification of secondary relaxation in glassformers based on dynamic properties. J. Chem. Phys..

[12] Capaccioli S, Prevosto D, Kessairi K, Lucchesi M, Rolla P (2007). Relation between the dispersion of α-relaxation and the time scale of β-relaxation at the glass transition. J. Non-Cryst. Solids.

[13] Capaccioli S, Kessairi K, Shahin Thayyil M, Prevosto D, Lucchesi M (2011). The Johari-Goldstein β-relaxation of glass-forming binary mixtures. J. Non-Cryst. Solids.

[14] Ngai KL (1998). Relation between some secondary relaxations and the α-relaxations in glass-forming materials according to the coupling model. J. Chem. Phys..

[15] Paluch M, Knapik J, Wojnarowska Z, Grzybowski A, Ngai KL (2016). Universal behavior of dielectric responses of glass formers: Role of dipole-dipole interactions. Phys. Rev. Lett..

[16] Jedrzejowska A, Ngai KL, Paluch M (2016). Modifications of structure and intermolecular potential of a canonical glassformer: Dynamics changing with dipole-dipole interaction. J. Phys. Chem. A.

[17] Jedrzejowska A, Wojnarowska Z, Adrjanowicz K, Ngai KL, Paluch M (2017). Toward a better understanding of dielectric responses of van der Waals liquids: The role of chemical structures. J. Chem. Phys..

[18] Sahra M, Thayyil MS, Bansal AK, Ngai KL, Sulaiman MK, Shete G, Hussan S (2019). Dielectric spectroscopic studies of three important active pharmaceutical ingredients—Clofoctol, droperidol and probucol. J. Non-Cryst. Solids.

[19] Körber T, Stäglich R, Gainaru C, Böhmer R, Rössler EA (2020). Systematic differences in the relaxation stretching of polar molecular liquids probed by dielectric vs magnetic resonance and photon correlation spectroscopy. J. Chem. Phys..

[20] Gainaru C (2019). Spectral shape simplicity of viscous materials. Phys. Rev. E.

[21] Pabst F, Helbling A, Gabriel J, Weigl P, Blochowicz T (2020). Dipole-dipole correlations and the Debye-process in the dielectric response of non-associating glass forming liquids. Phys. Rev. E.

[22] Pabst F, Gabriel JP, Böhmer T, Weigl P, Helbling A, Richter T, Zourchang P, Walther T, Blochowicz T (2021). Generic structural relaxation in supercooled liquids. J. Phys. Chem. Lett..

[23] Ngai KL (2011). Relaxation and Diffusion in Complex Systems.

[24] Ngai KL, Paluch M (2003). Inference of the evolution from caged dynamics to cooperative relaxation in glass-formers from dielectric relaxation data. J. Phys. Chem. B.

[25] Schneider U, Lunkenheimer P, Brand R, Loidl A (1998). Dielectric and far-infrared spectroscopy of glycerol. J. Non-Cryst. Solids.

[26] Gainaru C, Lips O, Troshagina A, Kahlau R, Brodin A, Fujara F, Rössler EA (2008). On the nature of the high-frequency relaxation in a molecular glass former: A joint study of glycerol by field cycling NMR, dielectric spectroscopy, and light scattering. J. Chem. Phys..

[27] Kessairi K, Capaccioli S, Prevosto D, Sharifi S, Rolla P (2007). Effect of temperature and pressure on the structural (a-) and the true Johari-Goldstein (b-) relaxation in binary mixtures. J. Non-Cryst. Solids.

[28] Blochowicz T, Rössler EA (2004). Beta relaxation versus high frequency wing in the dielectric spectra of a binary molecular glass former. Phys. Rev. Lett..

[29] P. Lunkenheimer, R. Wehn, U. Schneider, and A. Loidl, *Glassy aging dynamics*. Phys. Rev. Lett. **95**, 055702 (2005) 10.1103/PhysRevLett.95.055702 16090889

[30] Lunkenheimer P, Wehn R, Loidl A (2006). Dielectric spectroscopy on aging glasses. J. Non-Cryst. Solids.

[31] Li X, Wang M, Liu R, Ngai KL, Tian Y, Wang L-M, Capaccioli S (2015). Secondary relaxation dynamics in rigid glass-forming molecular liquids with related structure. J. Chem. Phys..

[32] Adichtchev SV, Bagdassarov N, Benkhof S, Blochowicz T, Novikov VN, Rössler EA (2002). Evolution of the dynamic susceptibility of paradigmatic glass formers below the critical temperature Tc as revealed by light scattering. J. Non-Cryst. Solids.

[33] Thayyil MS, Ngai KL, Prevosto D, Capaccioli S (2015). Revealing the rich dynamics of glass-forming systems by modification of composition and change of thermodynamic conditions. J. Non-Cryst. Solids.

[34] Ngai KL, Capaccioli S (2015). Reconsidering the dynamics in mixtures of methyltetrahydrofuran with tristyrene and polystyrene. J. Phys. Chem. B.

[35] Qi F, El Goresy T, Böhmer R, Döß A, Diezemann G, Hinze G, Sillescu H, Blochowicz T, Gainaru C, Rössler E, Zimmermann H (2003). Nuclear magnetic resonance and dielectric spectroscopy of a simple supercooled liquid: 2-methyl tetrahydrofuran. J. Chem. Phys..

[36] Pawlus S, Paluch M, Sekula M, Ngai KL, Rzoska SJ, Ziolo J (2003). Changes in dynamic crossover with temperature and pressure in glass-forming diethyl phthalate. Phys. Rev. E.

[37] Wagner H, Richert R (1997). Dielectric relaxation of the electric field in poly(vinyl acetate): A time domain study in the range 10^-3^–10^6^ s. Polymer.

[38] Richert R, Wagner H (1998). The dielectric modulus: Relaxation versus retardation. Solid State Ionics.

[39] Paluch M, Roland CM, Best A (2002). Dielectric and mechanical relaxation of cresolphthalein–dimethylether. J. Chem. Phys..

[40] Kahle S, Gapinski J, Hinze G, Patkowski A, Meier G (2005). A comparison of relaxation processes in structurally related van der Waals glass formers: The role of internal degrees of freedom. J. Chem. Phys..

[41] Casalini R, Paluch M, Roland CM (2002). Correlation between the α relaxation and the excess wing for polychlorinated biphenyls and glycerol. J. Thermal Anal. Calorim..

[42] Plazek DJ, Bero CA, Chay I-C (1994). The recoverable compliance of amorphous materials. J. Non-Cryst. Solids.

[43] Rizos A, Fytas G, Lodge TP, Ngai KL (1991). Solvent rotational mobility in polystyrene/aroclor and polybutadiene/aroclor solutions. II. A photon correlation spectroscopic study. J. Chem. Phys..

[44] Kahlau R, Dörfler T, Rössler EA (2013). Secondary relaxations in a series of organic phosphate glasses revealed by dielectric spectroscopy. J. Chem. Phys..

[45] Saini MK, Ngai KL, Jin X, Wang L-M (2020). Change in molecular dynamics with structures of the trialkyl phosphates and in mixtures with ortho-terphenyl. J. Non-Cryst. Solids.

[46] Sekula M, Pawlus S, Hensel-Bielowka S, Ziolo J, Paluch M, Roland CM (2004). Structural and secondary relaxations in supercooled di-n-butyl phthalate and diisobutyl phthalate at elevated pressure. J. Phys. Chem. B.

[47] Kudlik A, Benkhof S, Blochowicz T, Tschirwitz C, Rössler E (1999). The dielectric response of simple organic glass formers. J. Mol. Struct..

[48] Jakobsen B, Niss K, Olsen NB (2005). Dielectric and shear mechanical alpha and beta relaxations in seven glass-forming liquids. J. Chem. Phys..

[49] Corezzi S, Beiner M, Huth H, Schroeter K, Capaccioli S, Casalin R, Fioretto D, Donth E (2002). Two crossover regions in the dynamics of glass forming epoxy resins. J. Chem. Phys..

[50] Nielsen AI, Christensen T, Jakobsen B, Niss K, Olsen NB, Richert R, Dyre JC (2009). Prevalence of approximate√t relaxation for the dielectric α process in viscous organic liquids. J. Chem. Phys..

[51] Comez L, Fioretto D, Palmieri L, Verdini L, Rolla PA, Gapinski J, Pakula T, Patkowski A, Steffen W, Fischer EW (1999). Light-scattering study of a supercooled epoxy resin. Phys. Rev. E.

[52] Mierzwa M, Pawlus S, Paluch M, Kaminska E, Ngai KL (2008). Correlation between primary and secondary Johari-Goldstein relaxations in supercooled liquids: Invariance to changes in thermodynamic conditions. J. Chem. Phys..

[53] Hensel-Bielowka S, Ziolo J, Paluch M, Roland CM (2002). The effect of pressure on the structural and secondary relaxations in 1,1′-bis p-methoxyphenyl cyclohexane. J. Chem. Phys..

[54] Meier G, Gerharz B, Boese D, Fischer EW (1991). Dynamical processes in organic glassforming van der Waals liquids. J. Chem. Phys..

[55] Patkowski A, Gapinski J, Meier G (2004). Dynamics of supercooled van der Waals liquid under pressure. A dynamic light scattering study. Colloid Polym. Sci..

[56] Casalini R, Paluch M, Roland CM (2003). Influence of molecular structure on the dynamics of supercooled van der Waals liquids. Phys. Rev. E.

[57] Hecksher T, Olsen NB, Nelson KA, Dyre JC, Christensen T (2013). Mechanical spectra of glass-forming liquids. I. Low-frequency bulk and shear moduli of DC704 and 5-PPE measured by piezoceramic transducers. J. Chem. Phys..

[58] Blochowicz, T. *Communication on International Dielectric Society Meeting 2020*. and to be published.

[59] Richert R, Duvvuri K, Duong L-T (2003). Dynamics of glass-forming liquids. VII. Dielectric relaxation of supercooled tris-naphthylbenzene, squalane, and decahydroisoquinoline. J. Chem. Phys..

[60] Paluch M, Roland CM, Pawlus S (2002). Temperature and pressure dependence of the a-relaxation, in polymethylphenylsiloxane. J. Chem. Phys..

[61] Boese D, Momper B, Meier G, Kremer F, Hagenah JU, Fischer EW (1989). Molecular dynamics in poly(methylphenylsi1oxane) as studied by dielectric relaxation spectroscopy and quasielastic light scattering. Macromolecules.

[62] Gabriel JP, Zourchang P, Pabst F, Helbling A, Weigl P, Böhmer T, Blochowicz T (2020). Intermolecular cross-correlations in the dielectric response of glycerol. Phys. Chem. Chem. Phys..

[63] Wagner H, Richert R (1999). Equilibrium and non-equilibrium type beta-relaxations: D-sorbitol versus o-terphenyl. J. Phys. Chem. B.

[64] Leon C, Ngai KL (1999). Rapidity of the change of the Kohlrausch exponent of the r-relaxation of glass-forming liquids at T_B_ or T_β_ and consequences. J. Phys. Chem. B.

[65] Fytas G, Dorfmüller T, Wang CH (1983). Pressure- and temperature dependent homodyne photon correlation studies of liquid o-terphenyl in the supercooled state. J. Phys. Chem..

[66] Zhu XR, Wang CH (1986). Homodyne photon-correlation spectroscopy of a supercooled liquid: 1,3,5-tri-α-naphthyl benzene. J. Chem. Phys..

[67] Plazek DJ, Magill JH (1996). Physical properties of aromatic hydrocarbon. I. Viscoelastic behavior of 1,3,5-tri-alpha-naphthyl benzene. J. Chem. Phys..

[68] Ngai KL, Paluch M (2017). Corroborative evidences of TVγ-scaling of the α-relaxation originating from the primitive relaxation/JG β relaxation. J. Non-Cryst. Solids.

[69] Capaccioli S, Paluch M, Prevosto D, Wang L-M, Ngai KL (2012). Many-body nature of relaxation processes in glass-forming systems. J. Phys. Chem. Lett..

[70] Ngai KL, Paluch M, Rodríguez-Tinoco C (2017). Why is surface diffusion the same in ultrastable, ordinary, aged, and ultrathin molecular glasses?. Phys. Chem. Chem. Phys..

[71] Ngai KL, Valenti S, Capaccioli S (2019). Molecular dynamic in binary mixtures and polymer blends with large difference in glass transition temperatures of the two components: A critical review. J. Non-Cryst. Solids.

[72] Wang B, Zhou ZY, Guan PF, Yu HB, Wang WH, Ngai KL (2020). Invariance of the relation between α relaxation and β relaxation in metallic glasses to variations of pressure and temperature. Phys. Rev. B.

[73] Ngai KL (2020). Accounts of the changes in dynamics of hydrogen-bonded materials by pressure, nanoconfinement, and hyperquenching. Phys. Rev. E.

[74] Körber T, Minikejew R, Pötzschner B, Bock D, Rössler EA (2019). Dynamically asymmetric binary glass formers studied by dielectric and NMR spectroscopy. Eur. Phys. J. E.

