# Supplementary material for: Comparative analysis of dielectric, shear mechanical and light scattering response functions in polar supercooled liquids
Source: Sci Rep. 2021 Nov 12;11:22142. doi: 10.1038/s41598-021-01191-9 (PMC8589972; doi:10.1038/s41598-021-01191-9)
Supplement: Supplementary file 1 — Supplementary Information. [file 41598_2021_1191_MOESM1_ESM.docx]

**Comparative analysis of dielectric, shear mechanical and light**

**scattering response functions in polar supercooled liquids**

K.L. Ngai^1^, Z. Wojnarowska^2^, M. Paluch^2^

*^1^Dipartimento di Fisica, CNR-IPCF, Università di Pisa, Largo Bruno Pontecorvo 3, I-56127, Pisa, Italy*

*^2^Institute of Physics, University of Silesia in Katowice, 75 Pułku Piechoty 1A, 41–500 Chorzów, Poland*


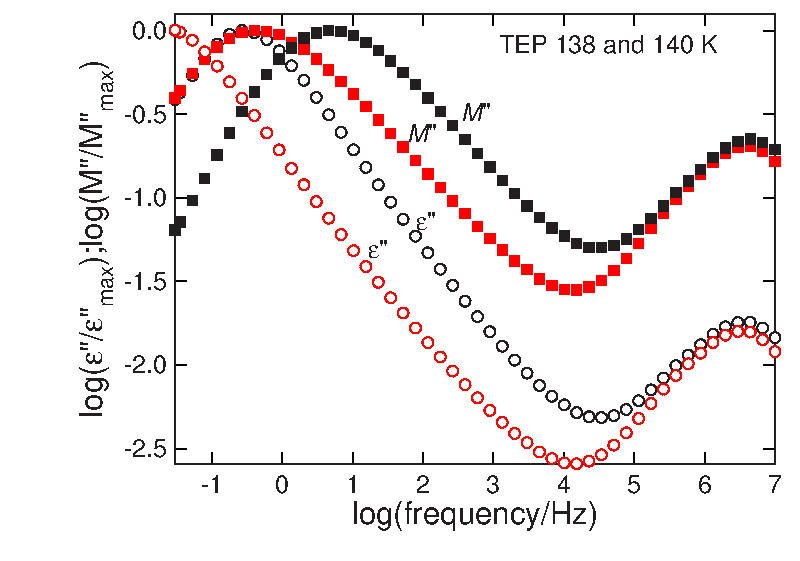


**Figure S1** The rescaled dielectric data of TEP recorded at 138 and 140 K. The data are taken from ref. R. Kahlau, T. Dorfler, and E. A. Rossler, *Secondary relaxations in a series of organic phosphate glasses revealed by dielectric spectroscopy*, J. Chem. Phys. **139**, 134504 (2013).


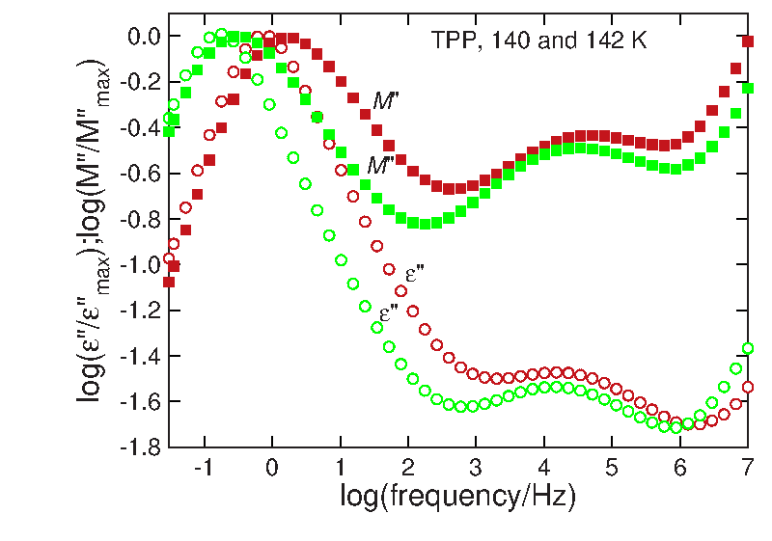


**Figure S2** The rescaled dielectric data of TPP recorded at 140 and 142 K. The data are taken from ref. R. Kahlau, T. Dorfler, and E. A. Rossler, *Secondary relaxations in a series of organic phosphate glasses revealed by dielectric spectroscopy*, J. Chem. Phys. **139**, 134504 (2013).
